# Visualizing and Quantifying Rembrandt’s Smalt Mixtures in *The Night Watch* in Three Dimensions Using μ‑X-ray Fluorescence Tomography

**DOI:** 10.1021/acs.analchem.5c07178

**Published:** 2026-09-17

**Authors:** Fréderique T. H. Broers, Annelies van Loon, Jan Garrevoet, Francesca Gabrieli, Jorien R. Duivenvoorden, Victor Gonzalez, Paul J. C. van Laar, Petria Noble, Koen Janssens, Florian Meirer, Katrien Keune

**Affiliations:** † Van’t Hoff Institute for Molecular Sciences, 365304 University of Amsterdam, Science Park 904, 1090 GD Amsterdam, The Netherlands; ‡ Science Department, Conservation & Science, Rijksmuseum, Hobbemastraat 22, 1071 ZC Amsterdam, The Netherlands; § Antwerp X-ray Imaging and Spectroscopy Laboratory,University of Antwerp, Groenenborgerlaan 171, 2020 Antwerp, Belgium; ∥ Inorganic Chemistry & Catalysis, Institute for Sustainable and Circular Chemistry, Utrecht University, Universiteitsweg 99, 3584 CG Utrecht, The Netherlands; ⊥ Photon Science at Deutsches Elektronen-Synchrotron DESY, Hamburg 22607, Germany; # Université Paris-Saclay, ENS Paris-Saclay, CNRS, PPSM, 4 Av. des Sciences, 91190 Gif-sur-Yvette, France; 7 Hamilton Kerr Institute and Conservation and Science Division, Fitzwilliam Museum, University of Cambridge, Trumpington Street, Cambridge CB2 1RB, United Kingdom; 8 Department of Conservation & Restoration, VICARTE, NOVA Universidade de Lisboa, 2829-516 Caparica, Portugal

## Abstract

Smalt is one of the main blue pigments in Rembrandt van Rijn’s *The Night Watch* (1642). Smalt is a cobalt-containing potash glass, ground to a powder to be used as an artists’ pigment. In this study, three smalt-containing paint samples from this painting were studied using synchrotron radiation-based micro-X-ray fluorescence (SR-μ-XRF) tomography. To study the original intentions of the artist, the degradation phenomena taking place in the paint and the best preservation methods for the painting, a fuller understanding of the smalt-containing paint mixtures is desired. The smalt particles in Rembrandt’s paints show a wide variety in size and elemental composition. Elements related to smalt are, e.g., cobalt, potassium, arsenic, bismuth, nickel, and iron. The heterogeneity is very high among the particles within one paint sample. Different clusters of particles can be established based on the intensity of bismuth. This variety could be caused by heterogeneity in the cobalt ore, the glass-making process, the use of multiple types of smalt or it might be the result of *in-situ* degradation. Next to the smalt, the volume percentages taken up by other components of the paint are semi quantitively estimated based on elemental information. This information can be used in follow-up studies to improve the accuracy of models and the representativeness of reconstructed paint mockup samples. SR-μ-XRF tomography proves to be a valuable tool for the identification and quantification of complex heterogenous smalt paints.

## Graphical abstract

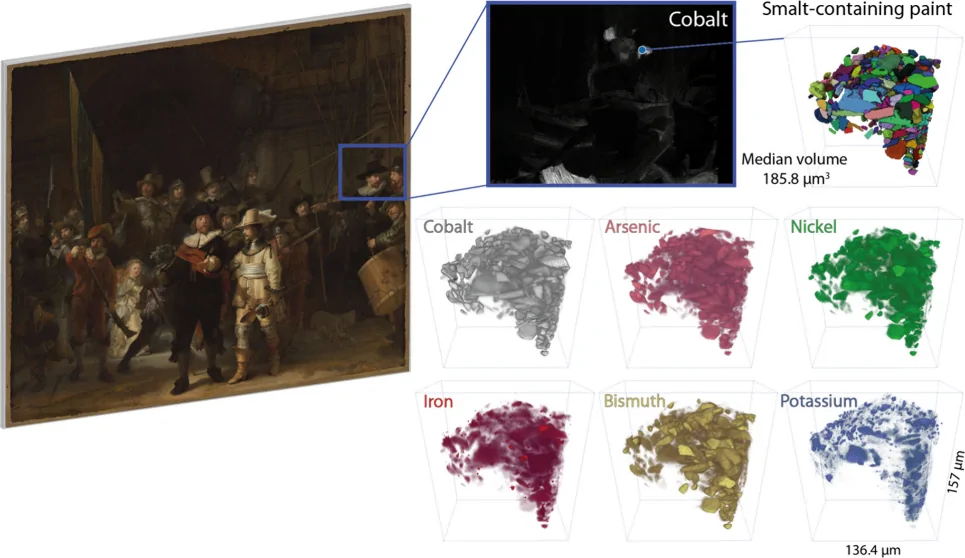

## Introduction

Historical oil paintings are both invaluable and vulnerable. To make sure we can share the joy of experiencing these paintings with future generations, it is necessary to preserve them, which requires a deep understanding of both material composition and current condition of the paintings. Here, we focus on the works of Rembrandt van Rijn (1606 Leiden–1669 Amsterdam, The Netherlands), one of the most well-known Dutch painters of the 17th century. More specifically, we studied the use and the degree of degradation of smalt, a commonly-used blue glass pigment in Rembrandt’s oeuvre.^1–9^ The smalt pigment was also used in the largest surviving painting of Rembrandt, *The Night Watch* (1642, Rijksmuseum Amsterdam). This masterpiece was studied with a wide variety of techniques as part of *Operation Night Watch*,^10–14^ the largest research and conservation project ever undertaken on this painting. In the current study, we used synchrotron radiation-based micro-X-ray fluorescence tomography to further our knowledge on the different smalt-containing paint mixtures that are present in *The Night Watch*.

Smalt is a finely ground, cobalt-colored blue potassium glass. Cobalt-bearing mineral compounds served as the primary precursor for smalt production in the 17th century. The initial step of the pigment synthesis involved the selection and purification of cobalt ores to obtain high-quality cobalt oxide. Different ores were used, containing minerals such as smaltite (Co, Ni)­As_3‑2_, erythrite (Co, Ni)_3_(AsO_4_)_2_·8H_2_O and cobaltite (Co,Fe)­AsS.^1,15,16^ Various methods were employed for the selection and purification, including roasting, grinding, washing and levigation of the ores. These first process steps were intended to eliminate impurities from the cobalt ore, such as arsenic, sulfur, and copper compounds, which could limit the pigment’s color purity and stability.^17–19^ Some recipes mention that non-treated ores were used.^20^ The resulting residue was mixed with a small amount of sand or quartz. This mixture is called *zaffre* or *zaffer­(a)*.^17–19^ The fusion of *zaffre* with powdered glass frit or silica and a fluxing agent formed the basis of smalt production. The silica or glass serves as network former to encapsulate the cobalt ions that give the resultant pigment its characteristic blue hue, while the flux, usually potash (an impure form of potassium carbonate), was added to reduce the melting temperature of the mixture.^21,22^ The exact composition of the glass plays a role in determining the final color and quality of smalt, with a higher cobalt-content resulting in more intensely colored glass. Once the *zaffre*, potash, and glass or silica were combined in powdered form, the mixture was subjected to intense heat through firing in a kiln. The controlled heating process was aimed at melting the glass, hereby incorporating the Co^2+^ ions into the glass matrix. Generally, no Co^3+^ ions are expected to be present if the alkali content is below 40 wt %, as these ions are not stable at the temperatures necessary for the fusion process.^23,24^ After cooling, achieved by quenching the hot blue glass mass directly into cold water, the resulting pieces were crushed into fine particles to yield the desired smalt pigment powder. The crushing and grinding process required precision and expertise to achieve the desired particle size, which directly influence the pigment’s color saturation and dispersibility.^16,25^ Different types of smalt, characterized by various levels of cobalt content, as well as smalt with varying grades of fineness were available in the 17th century, and it is likely artists used different types for different purposes.^6,8,26^ Smalt grades with a lower cobalt content were used e.g. for tinting of paper, while higher grades were usually employed in oil paintings.^17,24^

In many instances, smalt in oil paint mixtures has been reported to discolor over time. The phenomenon was already mentioned by contemporary 17^th^ century authors;^27^ it was studied more closely from the late 1960’s onwards by several authors,^15,28,29^ and it has been further investigated since then.^30,2,24,31–35^ In 1969, Plesters formulated different hypotheses for the discoloration of smalt.^28^ First of all, smalt has a low refractive index (RI, 1.47–1.55), close to that of fresh linseed oil (1.484); as linseed oil dries and ages, its RI increases to a value above that of the pigment. When this happens, there is less scattering of incident light by the pigment particles then by the oil matrix causing the paint layer to obtain a dark­(er) appearance, as there is not enough light scattering at the particular wavelength of the pigment color. Also, the yellowing of the oil could have a more important effect on smalt paint layers than on paint based on other pigments, due to smalt’s low RI. The other hypotheses are that either the alkali content of smalt or the phenomenon of cobalt leaching causes chemical reactions involving the oil or resin that surrounds the particles. The lower stability of potash glass compared to soda glass^36^ is considered to have an detrimental influence on the deterioration of smalt. Already in 1970, Giovanoli and Mühlethaler suggest, based on reflectance spectra recorded from unaltered and altered smalt, that the former contains cobalt ions in a tetrahedral coordination, while in the discolored smalt the cobalt ions are in an octahedral coordination.^29^ This change in coordination of the Co and O atoms results in the loss of the blue color of smalt. The same authors also report on the alteration of the surface of degraded smalt particles. Another very important piece of the smalt degradation puzzle was solved in 2001 when it was demonstrated that in discolored smalt particles, significant amounts of potassium had leached out.^30^ The same phenomenon was observed in a study in 2005 by Spring *et al*.^32^ In 2011, a synchrotron-based XANES study confirmed that the leaching of potassium out of the smalt particles causes an instability due to the fact that Co^2+^ ions are changing from a tetrahedral coordination to an octahedral coordination, resulting in a color loss. Often the concentration of potassium is higher in the core of discolored pigment particles and some studies show the presence of partially degraded smalt particles; in such cases the particles retain a blue colored core with original/higher levels of K and a discolored edge with lower/no concentration of potassium.^6,24,30,32^

Next to – or simultaneously with – the chemical changes in smalt particles, physical alterations can be observed over time as well. Microcracks or crizzling of the glassy particles have been reported to be present in degraded smalt.^20,37,16^ The cause(s) of these phenomena are probably directly related to the discoloration and the change in structure that takes place.^16^ The structural change at the surface of the particles leads to weathered or corroded surfaces. Manship et al. describe *cracking* and *corrosion* as two distinct phenomena, where the first phenomenon is characterized by sharply defined cracks and the second is recognized by a disruption of the surface, increased roughness and ‘wavy edges’.^38^

As smalt is the most important blue pigment used in *The Night Watch*, it is important to study the use of the pigment by Rembrandt as well as the current condition of the smalt. In some of Rembrandt’s paintings, various types or grades of smalt were found.^6,9,24^ In addition, Rembrandt is known to have used smalt in combination with other pigments, such as yellow or red lakes (organic dyestuffs precipitated with a mordant or binder), bone black (a black pigment obtained by calcination of animal bones, mainly composed of hydroxyapatite (Ca_10_(PO_4_)_6_(OH)_2_), earth pigments [in Rembrandt’s oeuvre mostly yellow (goethite, FeOOH) or red (hematite, Fe_2_O_3_) ochre] or lead white [a mixture of the lead carbonates cerussite (PbCO_3_) and hydrocerussite (Pb_3_(CO_3_)_2_(OH)_2_)].^2,6,8,39^ In some cases, the smalt was added to a paint not for its color, but for its drying properties or to provide texture or translucency to the paint.^7,8,16,40^

Knowing more about the smalt particles, the paint mixtures it is used in and its degree of degradation helps us to understand better the creative process of Rembrandt and the current condition of his paintings; it also allows to develop more realistic paint reconstructions or model systems for further studies. For this study, three paint samples containing smalt were selected from different areas in *The Night Watch*. The overall presence of smalt in the painting could be deduced from the cobalt distribution, detected by macroscale X-ray fluorescence (MA-XRF) scanning. Smalt was used extensively in the painting, both in the background and in the clothing. Co is a useful marker in XRF maps for smalt as it is the only pigment known in Rembrandt’s palette containing a considerable amount of Co. The presence of Co in historical paintings can also be related to modern pigments such as cobalt blue, which could be present from past restoration campaigns. There are no indications that this is the case in *The Night Watch*.

The three paint samples studied here with SR-μ-XRF tomography were selected from different areas in *The Night Watch* (see Figure 1 for their locations). Sample SK-C-5-043a (henceforth called sample **43a**) was taken in an area close to the upper right edge of the painting, from the arch from where the militia company is starting its march. As can be seen from the map (red rectangle), this area, at a height of more than 3.5 meters, was not painted in a detailed manner. This sample represents the use of smalt in the dark background paint. Sample **43a** consists of two layers, the quartz-clay ground layer (discussed in more detail in a previous study^13^) with a smalt-containing paint layer on top. This small paint sample was mounted on a pin for the tomographic study.

**Figure 1:**
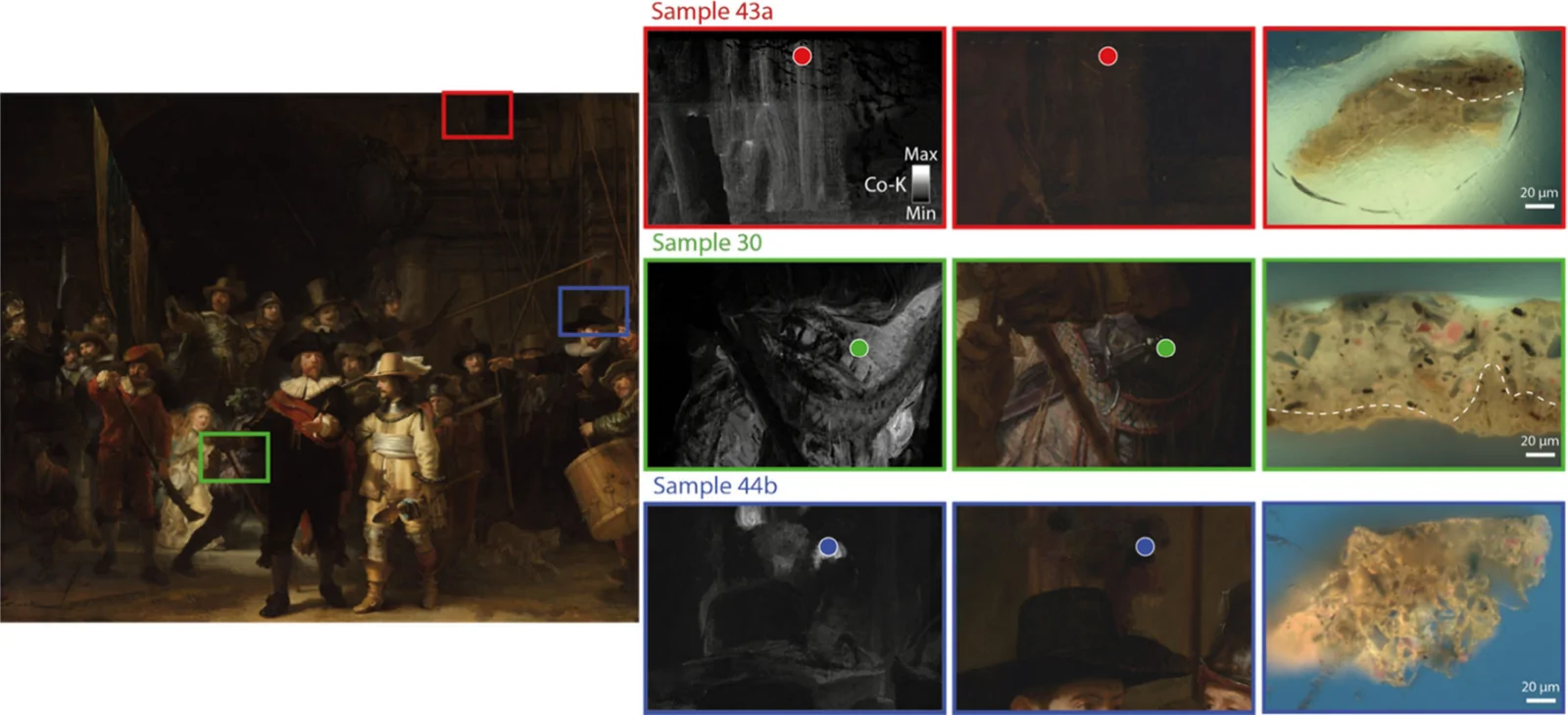
Rembrandt van Rijn, *The Night Watch*, 1642, oil on canvas, 410 cm × 485 cm. Rijksmuseum, Amsterdam, inv. no. SK-C-5 (artwork in the public domain). The inserts show the MA-XRF cobalt (K line) distribution maps of three areas from which samples were taken along with the images collected with 5 μm resolution photography of these areas.^42^ The locations of the samples are indicated with a dot in the insets for samples **43a** (red), **30** (green), and **44b** (blue). On the right, the samples as photographed under a 500× magnification and 365 nm UV light are shown. Sample **43a** and **44b** are photographed after embedding which was done after the 3D analysis. Sample **44b** is very thin in the area on the right, and some of the background color is seen through the transparent smalt particles. The white dashed lines are indicating the interface between the paint layer and the ground layer (lower layer).

The second sample studied was sample SK-C-5-030 (called sample **30** below). This sample is from the shadow area of the purple clothing of the shooting musketeer to the left of captain Banninck Cocq (the central figure of *The Night Watch*, in black with white collar and red sash).^39^ This sample mainly consists of the smalt-containing paint layer, but there is a very small area where some of the ground layer is present. Sample **30** was embedded in Technovit^®^ LC 2000 (embedding resin) as paint cross section.^41^

Sample SK-C-5-044b (called sample **44b** below) was taken just above the head of Sgt. Rombout Kemp from one of the purple feathers on his hat. (Kemp is the figure in a black costume with a white collar, wearing a black hat on the right side of the painting). The sample location is in one of the feathers in this area that contains elevated levels of cobalt. The sample was mounted on a pin for this study and consisted only of the smalt-containing paint layer.

Recently, the potential of synchrotron radiation-based X-ray fluorescence (SR-μ-XRF) tomography for the study of historical paint samples was demonstrated.^13^ Despite the foundational chemical insights provided by standard two-dimensional microscale analytical techniques, such as light microscopy (LM) or scanning electron microscopy (SEM), they struggle to capture the true volumetric heterogeneity of complex, multi-component matrices and inherently lack spatially resolved information in the third dimension. Therefore, a core motivating question underlying this work is how can we accurately and non-destructively quantify both the volumetric distribution and the elemental fingerprint of individual micro-particles embedded within dense, overlapping material systems? By utilizing SR-μ-XRF tomography, we overcome the structural flattening and spatial limitations inherent to standard 2D approaches, allowing us to isolate and analyze these particles in their true context. The data from the tomographic XRF experiment are used to generate a three-dimensional volume of the distribution of the detected elements. This 3D information can be used to accurately study the morphology of the paint, how different components relate to one another, as well as to quantitatively determine what kind of paint mixtures were used in an historical painting. Moreover, to assess the current condition of a painting, it is necessary to investigate ongoing degradation phenomena. In this study we will show how the SR-μ-XRF tomography can be used to answer research questions on the characteristics of the pigment smalt (mixtures) in *The Night Watch.* To answer the question whether different types of smalt were used, the distribution of elements present in or related to smalt are investigated. In addition, SR-μ-XRF tomography makes it possible to calculate the volume of the smalt particles and the particle size distribution, which is used to determine whether a difference in particle size is present among the available samples. Additionally, the presence and spatial distribution of other elements present in the samples helps to determine the composition of the paint mixtures in volume percentages that were used by Rembrandt in different areas of *The Night Watch*. The current condition of the smalt is studied to assess the smalt-related degradation phenomena taking place.

## Materials and Methods

### MA-XRF

Elemental maps of *The Night Watch* were generated using the commercially available MA-XRF scanner M6 Jetstream, manufactured by Bruker Nano GmbH (Berlin, Germany). This M6 Jetstream device is equipped with a 30 W rhodium-target microfocus X-ray tube, a polycapillary lens, and a dual 60 mm^2^ X-Flash silicon drift detector (SDD) with a beryllium window (energy resolution <145 eV at Mn Kα). The scanning head, mounted on a motorized X–Y stage, moves across the surface of the painting, allowing for an 80 × 60 cm^2^ scan area. Scanning was conducted at 50 kV and 200 μA with a step size of 500 μm and a dwell time of 35 ms per measurement.

To cover the entire painting, 56 overlapping scans (arranged in 8 rows and 7 columns) were performed, with ∼20% overlap horizontally and vertically. The M6 Jetstream was attached to a custom-designed platform (Bronnenberg) on scissor lifts. Throughout scanning, the instrument was carefully positioned to keep a consistent 10 mm distance from the X-ray source to the painting’s surface, maintaining a spot size of approximately 240 μm and minimizing fluctuations in X-ray fluorescence signal attenuation due to ambient air.

The spectral data cubes obtained from the MA-XRF scanning were analyzed using the software packages PyMCA (Python Multichannel Analyzer) and Datamuncher γ 1.4.^43,44^ A spectral fitting model was made in PyMCA. In the resulting 2D distribution maps, each pixel represents the calculated net peak intensities of the element’s emission lines, displayed in a grayscale proportional to the detected intensities.

### X-ray Fluorescence Tomography

Three samples (**43a**, **30**, and **44b**) were all scanned at beamline P06 (PETRA-III, DESY, Hamburg, DE) using a XRF tomography set-up with a step size of 200 nm.^45^ XRF data was collected by two Si Drift Vortex detectors.

The X-ray fluorescence tomography experiments were conducted at beamline P06 at PETRA III, DESY (Hamburg, Germany). Sample SK-C-5-030 was measured embedded in Technovit 2000 LC mounting resin (Heraeus Kulzer GmbH, Germany). The resin was thinned down to ∼200 μm to limit interference of the embedding medium. Samples SK-C-5-43a and -44b were directly positioned on a tip with epoxy glue. The embedded sample and the samples glued on a tip were mounted on a rotating stage. To obtain synchrotron X-ray fluorescence (SR-μ-XRF) data for each 2D projection, two XRF detectors were positioned perpendicularly on either side of the sample relative to the primary X-ray beam. In case of SK-C-5-030 (October 2020), two SII Vortex EM Si-drift detectors were used. Scans were performed at each degree from 0 to 180, then again from 180.5 to 360.5 degrees, totaling 362 scans. The primary X-ray beam operated at 17 keV, with a step size of 200 nm and a dwell time of 2 milliseconds per scan. Samples **43a** and **44b** (September 2021), were scanned using a SII Vortex EM Si-drift detector on one side and a SII Vortex ME4 Si-drift (4-element) detector on the other side. Scans were collected every 2 degrees from 0 to 178 and every 2 degrees from 181 to 359 degrees (total of 178 scans). The energy of the primary X-ray beam was 17 keV, a step size of 200 nm and a dwell time of 4 milliseconds per scan. The ME4 detector could not be operated at a dwell time of 2 milliseconds, therefor the dwell time had to be doubled in this set-up. The additional scanning time was compensated by reducing the number of scans.

Deconvolution of the generated XRF data was done by using a fitting model created in PyMCA.^46^

The XRF data were processed with a fitting model using PyMCA, which was applied to the entire data set. For each projection, images from the two detectors were combined. Some projections had pixels with unusually high intensities, which were corrected by replacing these values with the average of the surrounding pixels. For clusters of high-value pixels in certain elements, outlier values were replaced on the basis of a threshold derived from the 2D image histogram. In this way, 2D distribution maps were generated of the XRF data for all of the projection angles.

More information on the data processing framework at beamline P06 can be found in a conference paper by Garrevoet et al.^47^

### 3D Reconstruction

The fitted 2D projection images were used to create 3D reconstructions of the distribution of different elements present in the paint samples. The 3D reconstruction was performed using TXM Wizard software. TXM Wizard software was used to align a common feature across all projections, employing an iterative algebraic reconstruction (i-ART, i = 20) algorithm built-in into the software.^48^ For samples SK-C-5_043a, 177 projections were used, for sample SK-C-5_044b, 176 projections were used, and for SK-C-5_030, 362 projections were used.

The 3D reconstructions were further processed using the Avizo 3D software package.^49^ This software was used for segmentation and visualization. More details about the segmentation can be found in the Supporting information.

The 3D resolution was calculated to be 673 nm for sample **43a**, 916 nm for sample **30** and 670 nm for sample **44b** see the Supporting information, Figure S22 for more information.

### 2D μ-XRF and μ-XRD

The distribution maps of copper and azurite shown in Figure S18b,c were collected at beamline P06 at PETRA III, DESY (Hamburg, Germany). The energy of the beam was 17 keV. The map was collected with a step size of 1 × 1 μm^2^ and a dwell time of 0.25 seconds. XRD data was collected by an Eiger X 4M hybrid pixel detector. The XRF data was collected with a SII Vortex EM Si-drift detector and a SII Vortex ME4 Si-drift (4-element) detector. The XRD maps were processed with XRDUA.^50^

### Light Microscopy

Light microscopy images were taken using a Zeiss Axio Imager.A2m microscope fitted with a Zeiss AxioCam MRc5 digital imaging system. Observations were carried out at magnifications of up to 500× under both bright-field illumination and ultraviolet light conditions (UV-A, 365 nm), provided by a Zeiss Colibri 2 LED source. Luminescence images were recorded using a filter cube containing a 365 nm excitation filter (EX G 365), a 395 nm beam splitter (BS FT 395), and a 420 nm long-pass emission filter (EM LP 420).

### Scanning Electron Microscopy–Energy Dispersive X-ray Analysis (SEM–EDX)

The samples were analyzed using a FEI NovaNano SEM 450 scanning electron microscope. Elemental spectra were collected at 20 keV by the Thermo Scientific UltraDry EDS and the resulting data were processed using Thermo Scientific Pathfinder X-ray Microanalysis Software.

## Results

### Defining Smalt Particles: Cobalt Distribution and Smalt Particle Size

Figure 2 shows the distribution of cobalt in the three samples in two (Figure 2a–c) and three (Figure 2d–f) dimensions. The smalt particles, being ground glass particles, generally feature quite sharp edges. The virtual orthoslices in Figure 2a–c show these sharp edges as well as the differences in intensity in Co among the smalt particles in the samples. These slices, as well as the three-dimensional volume renderings of the cobalt signal in Figure 2d–f, show a difference in the amount of smalt present in the three samples, where **43a** has the lowest amount of smalt in the paint layer, followed by sample **30**, and sample **44b** has the highest amount of smalt. The differences in volume percentages are discussed later on. The volume renderings in Figure 2d–f also show that there is a wide variety in particle size.

**Figure 2:**
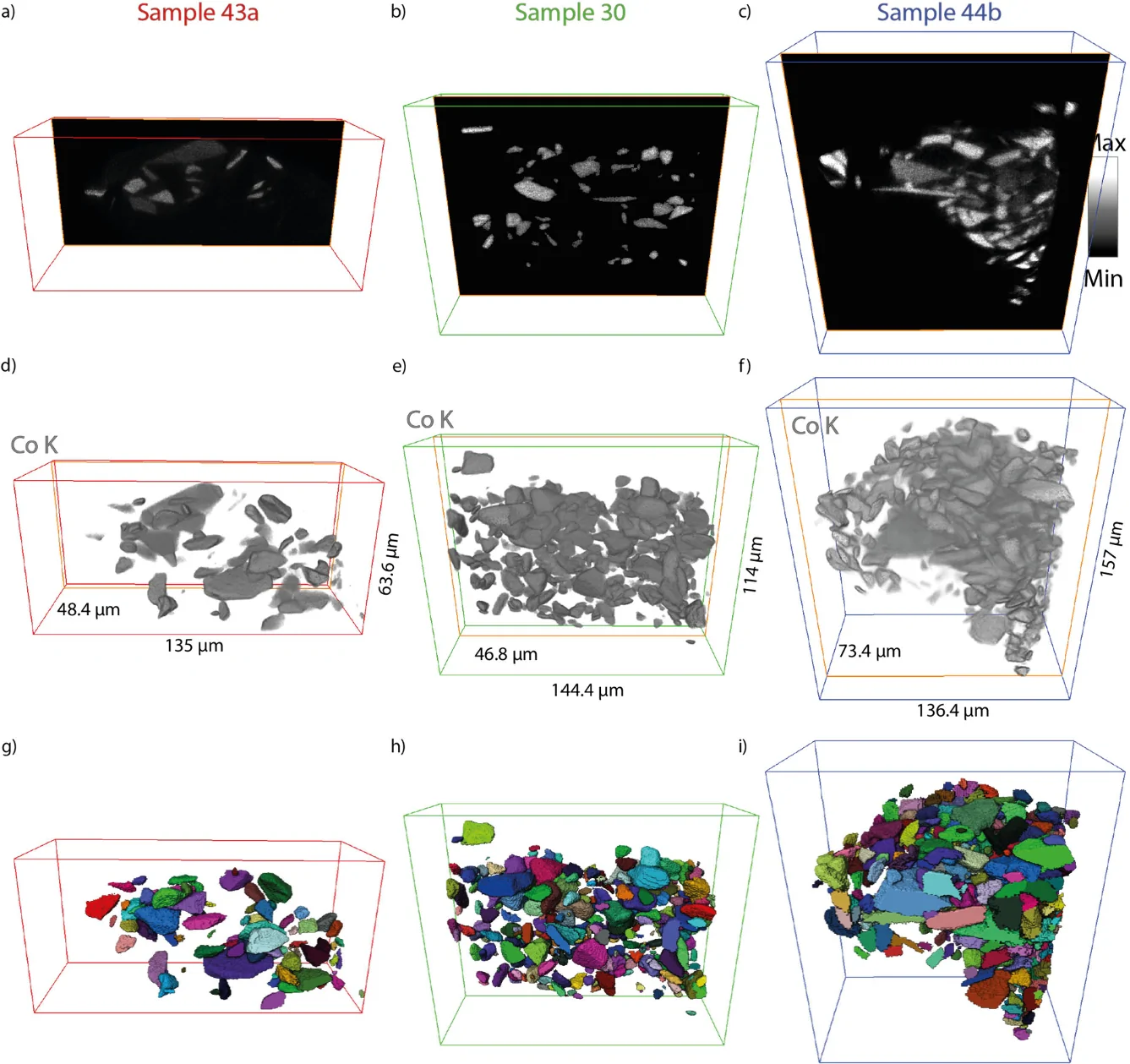
In (a–c), one virtual orthoslice of the 3D volumes reconstructed from XRF data are shown, showing the cobalt distribution in samples (a) **43a** (red), (b) **30** (green), and (c) **44b** (blue). All samples show a variety of particle sizes and shapes with lower and higher concentrations of cobalt. 3D volumes of the cobalt distribution in samples (d) **43a** (red), (e) **30** (green), and (f) **44b** (blue). The orange rectangles indicate the position of the orthoslices shown in (a–c). (g–f) Segmented smalt particles (based on Co–K distribution) of samples (g) **43a**, (h) **30**, and (i) **44b** in random colors. The samples are all oriented with the painting side facing up, and the canvas side towards the bottom.

To quantify the particle sizes, a careful segmentation is required. Although the human eye does not have a lot of trouble distinguishing separate particles, automating this segmentation is challenging. Especially in samples **30** and **44b**, where the smalt particles are very densely packed, contacting particles are not easily segmented properly. This happens for example when the segmentation is based only on a threshold value. This method segments particles based on a user-determined intensity value, or a value calculated based on the histogram. A method that has shown to improve segmentation for contacting particles is watershed segmentation. This algorithm creates lines based on the highest gradient that delineates class boundaries.^51,52^ By employing the built-in watershed algorithm in Avizo, we were able to improve the segmentation of contacting particles. As this method exploits the gradient in grey scale to define the boundaries of the particles, some artefacts can be introduced: for example, in the case of a crack or irregularity in the particles, the algorithm may recognize this as a formal edge and segment one smalt particle having cracks into multiple individual particles. On the contrary, some particles that are next to one another and have a very similar grey scale value (because their Co concentration is nearly the same) might still be improperly segmented. This problem was partly solved by manually removing some material at the interface of two particles and then employing the ‘Separate objects’ algorithm in Avizo. More information on the segmentation procedure can be found in the Supporting Information.

There is a considerable variety in the smalt particles sizes within every sample. Figure 2g–i shows the segmented smalt particles, with every separate particle in a different random color. The median, mean, minimum and maximum particle size of samples **43a**, **30** and **44b** are listed in Table 1. The particle size distribution of the samples can be found in the supporting information, Figures S1–S3. If we consider the median volume of the smalt particles, sample **30** has the smallest particles with an average of ∼62 μm^3^. Smalt particles in sample **43a** have a slightly larger median volume at ∼98 μm^3^. The particles in sample **44b** have a considerate larger median volume at ∼186 μm^3^. The large difference between the median and mean volume indicates that for all samples there is a large fraction of particles smaller than the mean, as can also be seen in the particle size distribution in Figures S1–S3. The large variety in particle dimensions also explains the large standard deviation (SD) in Table 1. Additional to the volume of the particle, the Feret 3D shape (*D*/*d*) can be used to inform on the shape of the particle. The Feret diameter is a measure to determine the diameter of an irregularly shaped particle. As we are dealing with three-dimensional particles, the minimum Feret diameter (*d*) was determined by calculating the diameter along 31 directions in the particle and selecting the smallest value. *D* is the maximum Feret diameter in the orthogonal directions (90 deg from *d*). In the case of perfect spherical particles, the Feret 3D shape (*D*/*d*) is 1; the larger this number, the more elongated (needle-like) the particle is. The Feret 3D shapes for all three samples are between 1.99 and 2.37 (see Table 1). In Table S1, the mean value of the maximum and minimum Feret diameters (calculated in Avizo) of the three samples can be found.

**Table 1:** Median, Mean, Minimum and Maximum Smalt Particle Size in Samples **43a**, **30**, and **44b** (Excluding All Clusters of 100 Voxels or Smaller)

	**43a**	**30**	**44b**
median volume	98 μm^3^	62 μm^3^	186 μm^3^
mean volume	189 μm^3^ (SD 279.1)	180 μm^3^ (SD 321.8)	429 μm^3^ (SD 877.6)

minimum particle volume ()	1.1 μm^3^	0.8 μm^3^	0.9 μm^3^
			

maximum particle volume	1587 μm^3^	2092 μm^3^	10,197 μm^3^
Feret 3D shape (D/d) (median)	2.37	2.12	1.99
number of particles	82	320	413

The large heterogeneity observed in the smalt particle size distribution within one sample shows the importance of studying as many particles as possible from an area before drawing any conclusions about the particle size variation among paint samples. One might argue that the heterogeneity within one area is so large that it is difficult to draw any meaningful conclusions from the average sizes.

### Elemental Distribution in Smalt Particles

As known from the literature, there are other elements apart from Co that are often found in smalt particles: arsenic (As), nickel (Ni), iron (Fe), bismuth (Bi), and potassium (K).^1,16^ Ni, As, Fe, and Bi are trace elements originating from the cobalt ore. Potassium is incorporated during the fusion process of the *zaffre* with either pre-fabricated potash glass frit, or as a flux for fusion with silica. Parts b–f of Figure 3 show the three-dimensional distribution of these elements for sample **44b** in comparison to that of Co (Figure 3a).

**Figure 3:**
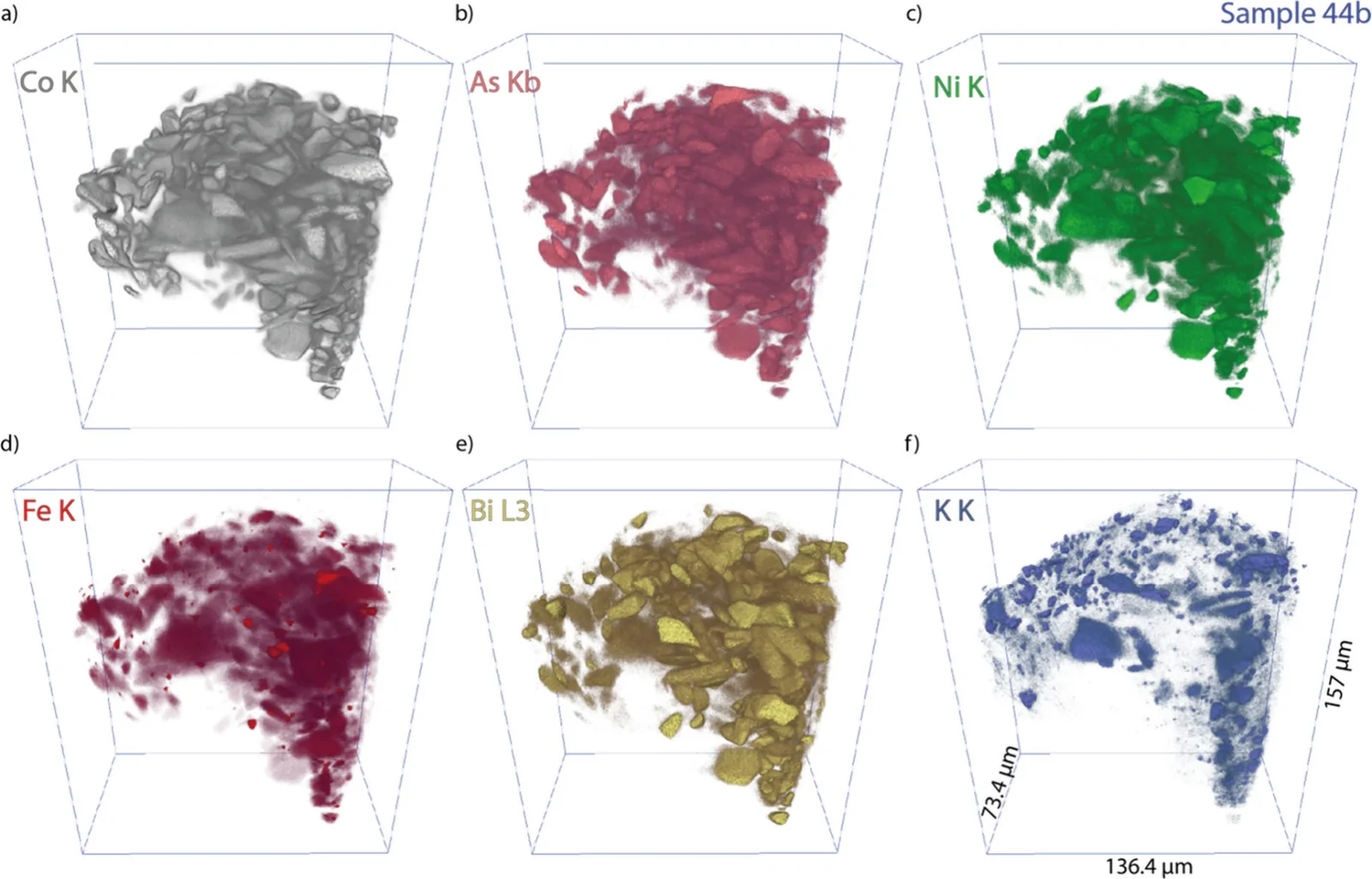
3D volumes of the distribution of (a) cobalt (blue), (b) arsenic (pink), (c) nickel (green), (d) iron (red), (e) bismuth (yellow), and (f) potassium (blue/purple) in sample **44b**. The lowest 10% of the histogram is not shown to avoid visualization of background noise.

In Figure 3, we see that the smalt particles observed in the Co distributions in part a) can also be distinguished in the As, Ni, Fe, and Bi distribution (parts b–e). The same trend can be seen within XRF volume maps of samples **43a** and **30** (see Figure S4 and S5, respectively): again, there is a clear correlation between these elements and the smalt particles. However, in sample **43a**, the Fe distribution map does not immediately show distinct smalt particle shapes. This is due to very concentrated iron-containing particles in the earth pigments in the paint and in the ground layer present in this sample, causing a different scaling of the image intensity. Also in Figure 3d, next to the smalt particles there is a clear presence of Fe in small particles around the smalt. Again, this can probably be related to the presence of earth pigments.

The quantitative assessment of the trace elements in smalt is difficult due to matrix absorption effects. The paint composition differs among the samples, and (re)­absorption and scattering of the primary and fluorescent X-rays both influence the detected yields. In addition, sample **30** was examined in an embedding medium, while **43a** and **44b** were mounted directly on a pin, which has an influence on the absorption of the (low-energy) X-rays. These factors make a quantitative comparison of the concentrations among the three samples impossible at this time. We will therefore investigate only the trends in correlation between the elements per sample.

### Chemical Fingerprint of Smalt Particles

To study differences between smalt particles within one sample, we investigated the characteristic elemental fingerprints of each separate smalt particle. For the individual particles shown in Figure 2g–i, the average intensity per element per particle is registered. This information was used to perform an agglomerative hierarchical clustering procedure. Figure 4 shows the average intensity for the clusters identified in the samples and their position in the sample. More information on the clustering can be found in the Supporting information.

**Figure 4:**
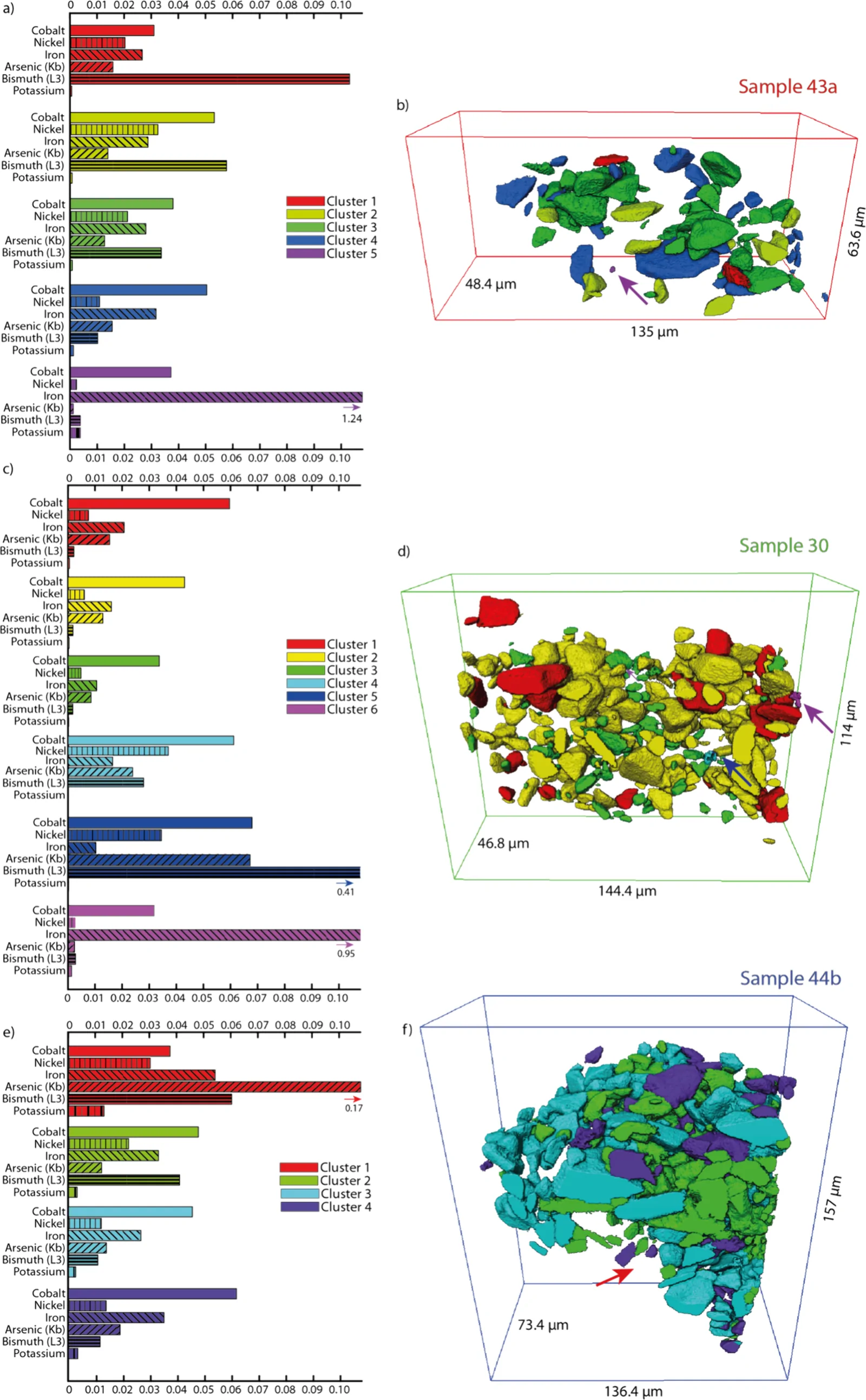
Elemental fingerprint of the clusters identified by agglomerative hierarchical clustering using the average intensity (a,c,e) and the volume rendering of the sample where the segmented smalt particles are colored based on the cluster they belong to (b,d,f). (a,b) The results for sample **43a**, (c,d) for sample **30**, and (e,f), for sample **44b**. The different elements in (a,c,e) can be recognized by the different patterns in the bar charts.

Based on the clustering result of sample **43a**, shown in Figure S6, five clusters were selected. Parts a and b of Figure 4 show the average intensity of the clusters and the location of the particles of the clusters in the sample. Cluster 5 (purple) is clearly an outlier with a very high intensity of Fe (Figure 4a). This particle has a very low concentration of all other elements except Co, indicating that it might be another Fe–Co component, possibly a residue from the ore. Two particles belonging to cluster 1 (red) were identified. These particles have a relatively high intensity of Bi compared to the particles belonging to the other clusters (Figure 4a). 9 out of the 82 particles in the sample belong to cluster 2 (yellow). This cluster has a relatively high intensity of Co, Ni, and Bi compared to the bulk of the particles. The remaining 70 particles are divided into clusters 3 (green) and 4 (blue). The particles belonging to cluster 4 have a very low intensity of Bi. Spatially, the particles belonging to the different clusters are dispersed through the sample (see Figure 4b). There does not seem to be a correlation between particle size and clusters (see Figure S9a for the average particle size per cluster).

In sample **30**, six clusters were identified (Figure S7 and Figure 4c,d). In clusters 4 (cyan), 5 (blue), and 6 (purple) outliers are grouped. Specifically, cluster 4 and 5 are related to the edges of a very intense Co-particle that was removed before segmentation of the smalt particles. This masking was done based on a manual thresholding, and some of the edges of the particle were not completely removed. These remnants are aggregated in clusters 4 and 5. The bulk of the particles belong to cluster 1 (red, 33 particles), 2 (yellow, 175 particles) and 3 (green, 101 particles). Cluster 3 has the relative lowest intensity of Co, cluster 2 has a medium intensity and cluster 1 has the highest intensity of Co. The Ni, Fe, and As intensity follow the same trend for the three clusters. The Bi intensity is low in all clusters, but the relative Co:Bi ratio in cluster 3 is the highest out of the three. The particles of cluster 1, 2, and 3 are evenly dispersed through the sample. The average particle size (Figure S9b), shows that the particles in cluster 3 are smaller than in the other clusters, with a median size of 19 μm^3^ versus 113 μm^3^ in cluster 2 and 188 μm^3^ in cluster 1.

Figure S8 and Figure 4e,f show the clusters identified in sample **44b**. Four clusters were obtained, with only one particle belonging to cluster 1 (red). Figure 4e shows that the intensity of As is extremely high in the single particle belonging to cluster 1. This particle is also characterized by a higher signal intensity for Ni, Fe, Bi, and K in comparison to the other smalt particles. This particle is not visible in the view in Figure 5c but can be seen in Figure S10. Cluster 2 (green) has an increased intensity of Bi in comparison with cluster 3 (cyan) and cluster 4 (purple). Cluster 4 has an increased intensity of Co and As in comparison with clusters 2 and 3. The variation in intensity per element per cluster can be found in Figure S11. The correlation plots of all elements can be found in Figure S12. Again, the particles belonging to the different clusters are heterogeneously dispersed throughout the sample. No clear correlation between particle size and a certain cluster was observed (see Figure S9c).

**Figure 5:**
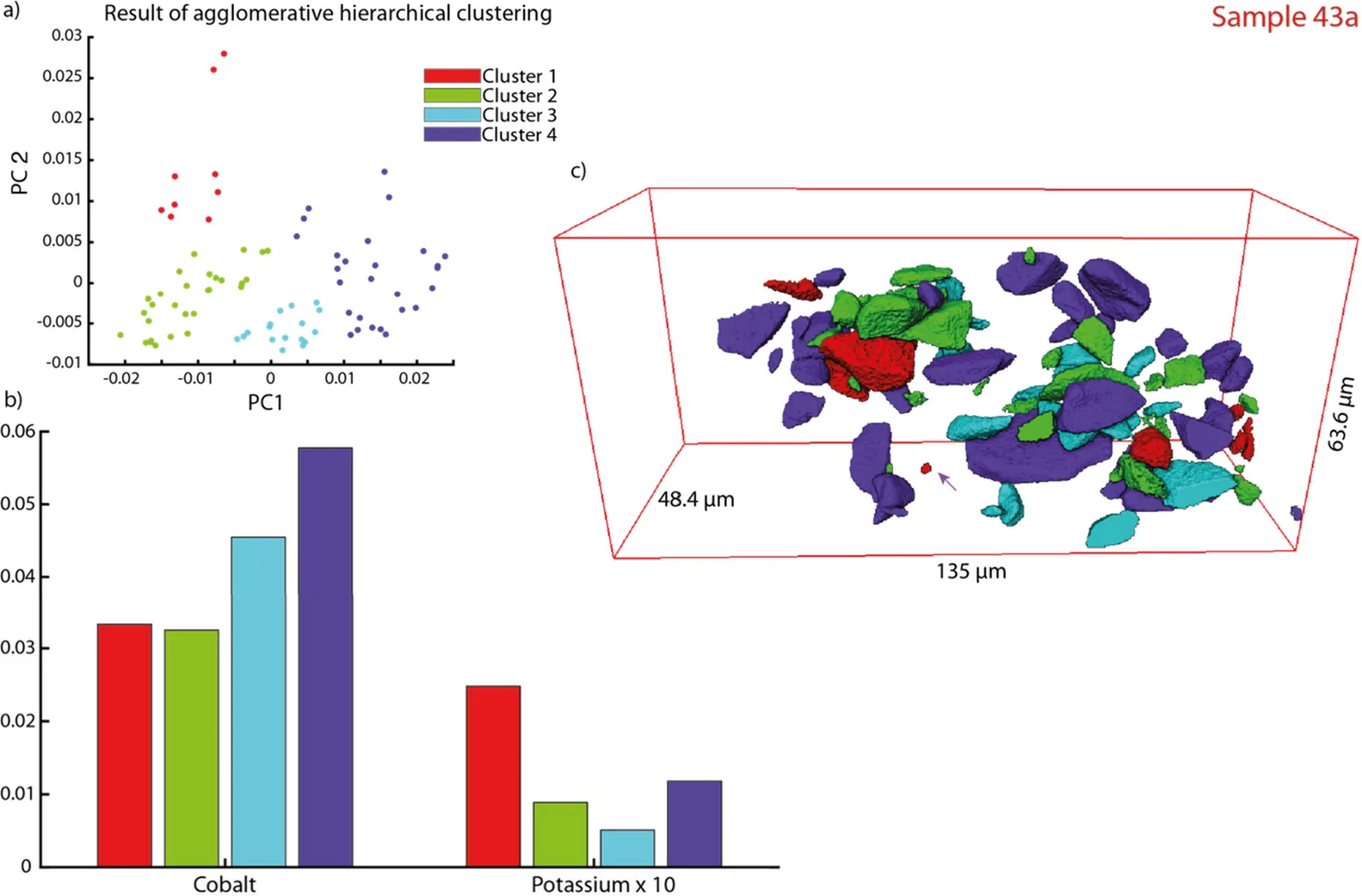
Clustering results of the smalt particles of sample **43a** based on their cobalt and potassium intensities. (a) The result of the agglomerative hierarchical clustering. (b) Bar plot of the average intensity of cobalt and potassium (multiplied by 10) over the four clusters. (c) Volume rendering of the sample, the segmented smalt particles are colored based on the cluster they belong to.

In all samples there is a variation in the Co:Bi ratio among the different particle clusters (although less pronounced in sample **30**). Most smalt studies in literature do not mention concentration trends of Bi, due to the low concentration of this minor element and the difficulty to detect it with SEM-EDX. Several hypotheses can be proposed to explain this variation in Co:Bi ratio:

(1) Several different types of smalt with different Co:Bi ratios were used. This could either be a deliberate choice of Rembrandt or the smalt could have been sold as powdered mixtures from different sources or batches. Not a lot is known in the literature about how the trade of cobalt ores or *zaffre*, e.g., from Saxony to The Netherlands, took place in the 17th century.^5,17,19^ It is also possible that the *zaffre* producers, or smalt producers in The Netherlands, mixed the products from different batches based on similarity in color, or that they mixed differently colored batches to create new hues.
(2) A second hypothesis is that the heterogeneity in Co:Bi ratio was present in the cobalt ore. In a study by Zlámalová et al. it was demonstrated that archeological smalt samples collected at one smalt production site in Bohemia show a considerable variety in Co:Bi ratio.^25^ Panighello et al. also report a variety in the Co:Bi ratio in smalt within two Dutch 17th century oil paintings.^53^ These authors also emphasize the heterogeneity of the cobalt ores and the inevitable heterogeneity this will cause in the resulting smalt.
(3) The variety in Co:Bi ratio among the particles could also be the result of the manufacturing process. As explained earlier, the ores were usually first roasted (up to 700 °C) to eliminate elements such as As and S. Literature does not mention if there is an effect on the Bi during this step. Then, during the melting of the glass and *zaffre* mixture, the mixture is exposed to even higher temperatures, up to 1100–1250 °C.^15,16,25^ This step might also have an effect on the element ratios in the mixture. With 17th-century technology one can imagine that the manufacturing process was not fully controllable, which might have resulted in an inhomogeneous product. Throughout the kiln that was used for the process, the temperatures may have varied, resulting in differences within one batch of smalt. More research is required on the potential compositional heterogeneity within individual smalt batches as a result of the variable production process.

A fourth explanation of the variety in Co:Bi could be related to the degradation of smalt, discussed in the next section.

Literature on smalt pigment often report the composition based on EDX measurements.^8,32,53–56^ EDX measurements on smalt particles in samples taken from the same areas as the 3D studied samples are provided in Tables S2–S4.^39^ The semi-quantitative EDX analysis again shows the presence of a large variety in composition of the smalt particles within one sample. Some of the largest variations are in the amount of potassium, explained by the presence of both degraded and nondegraded smalt particles (see also the next section). There are also large variations in the amounts of cobalt. The concentration of cobalt is mentioned in the literature as an indicator of the grade of smalt, with a higher concentration of cobalt representing a better grade and brighter blue smalt.^54^ To determine the concentration of CoO, it is necessary to use measurements acquired in well-preserved smalt particles. In both samples **43** and **44**, only one intact smalt particle was present, therefore, we are not able to report the average CoO concentration of these samples. To study these and related questions about the chemical composition of the smalt used in *The Night Watch* in more detail and to compare the smalt used in this painting with that of other Rembrandt paintings and those of contemporaries, a future study will focus on this topic and present SEM-EDX results on a larger number of samples from the painting.

### Degradation of Smalt

As mentioned, the most well-known degradation mechanism of smalt is related to the discoloration of the blue pigment to a grayish hue, linked with a phenomenon of potassium leaching from the particles. This degradation is not distributed equally across smalt grains, as partially degraded smalt particles are found frequently in works of art: these particles have a gradient of K concentration between the nondegraded core and the discolored edges of partially degraded smalt particles.^6,24^ Focusing on the potassium distribution in 3D in Figures 3 and S4, a higher concentration of potassium is observed in some of the cores of the particles in comparison to edges. In all samples, fewer smalt particles are observed in the potassium distribution compared to the other elements. This shows that some of the smalt particles do not contain any potassium (at a detectable level).

It should be noted that potassium is difficult to measure at the hard X-ray beamline P06 using XRF. Less signal is coming from the center of the sample compared to the surface due to self-absorption (absorption of the K–K radiation on its way to the XRF detector(s) by the sample itself). Especially in sample **30**, which was measured in an embedding medium, almost no K signals were detected in the center of the sample (see Figure S5).

Because of the low intensity of K in the XRF spectra recorded from the smalt particles, the differences in K intensity do not contribute as much as the other elements to the clustering calculations shown before. However, a clustering of the particles focused on the K intensity can still be performed by (artificially) adjusting the intensity ratio; in this case, it is done by multiplying the intensity values of potassium with a factor of ten. For this clustering, only Co and K information was used. The clustering is performed on the average intensity of the elements per particle and can therefore not show the gradient in K within the single particles. Figure 5 shows the results of the clustering for sample **43a** selecting four clusters. Clusters 1 (red) and 2 (green) have a comparable Co intensity, but the average intensity of K in cluster 1 is relatively much higher than that in cluster 2. Cluster 3 (cyan) has a higher average intensity of Co than 1 and 2 but the lowest K intensity of all clusters. The last cluster, cluster 4 (purple), has the highest Co intensity and a medium K intensity. These results show that, in accordance with the literature, there are small particles still high in K and particles from which most K has leached. There is no direct correlation between the intensities of Co and K. The particle sizes within clusters have a high variety (see Figure S13a), and there is no correlation between the intensity of K and the average particle size in the clusters.

In Figure S14 the results for the clustering with Co and K (multiplied by 10) of sample **44b** are shown. Again, in this sample, there is one cluster (4, purple) consisting of smalt particles still containing a high intensity of K. Cluster 3 (cyan) has a medium intensity of K and a high intensity of Co and clusters 1 (red) and 2 (green) mainly differ in their intensity of Co. The average particle sizes of the different clusters are very similar (see Figure S13b).

The SEM-EDX measurements on the cross sections (Tables S2–S4), also show that mostly potassium poor smalt particles are present in the paint samples. Better-preserved smalt particles, with a K_2_O concentration of 7% or higher measured at the core of the particle, are indicated with a black outline. The results show that sample **30** contains the most well-preserved smalt particles.

The elemental distribution in the previous figures in this study are presented in such a way that the pigment particles are clearly visible. This is done by excluding the lowest part (∼10%) of the histogram in the visualization. Figure S15a,b show the Co and Bi distribution in sample **43a** from the minimum to the maximum intensity, so the full histogram is shown. This view shows that apparently, low concentrations of both Co and Bi are present throughout the sample, as well as K (see Figure S15c). There is no correlation between low Bi levels and low K levels, as the particles belonging to cluster 4 (low Bi) in Figure 4a,b are not the same particles belonging to cluster 2 and 3 (low K) in Figure 5. The distribution of Ni and As is more restricted to the smalt particles (see Figure S11d,e). For Fe, the low concentration distribution is difficult to assess due to the presence of other Fe-containing components (mainly earth pigments) in the paint (Figure S11f). The leaching of Co from smalt particles has been suggested before,^34^ but there are also authors that claim there is no leaching of Co in smalt paint.^30^ The low detection limit of the current synchrotron-based study show that there are indications for the leaching of other elements than K, however in XRF tomography, background signal caused by the matrix of the sample can be misinterpreted as signal from a certain element when a fitting procedure is performed. Therefore, at this point it is difficult to asses if the variation in Co:Bi ratio could have arisen *in-situ* due to chemical alteration of the pigment and leaching of Co and/or Bi from the smalt particles. This phenomenon should be further studied with sensitive equipment, e.g., by degradation studies of simple, thin layered model systems of smalt paints at synchrotron radiation facilities.

Another degradation phenomenon observed in the samples is the formation of microcracks in some of the smalt particles. Figure S16 presents one of the smalt particles in sample **44b** that shows some cracks. The literature suggests that the formation of these cracks is related to the degradation of the glassy pigment.^16,20,37^

### Composition of Smalt Paint Mixtures

The 3D reconstructions of the XRF results give an insightful view of the multimaterial composition of the smalt paint mixtures. The differences in this respect among the three different paint samples can be explored by considering the volume maps of elements that are not related to smalt particles. Figure 6 and Movie S1 show the 3D volume rendering of the main elements present in sample **43a**. This sample consists of a smalt-containing paint layer (top) and the clay-quartz ground layer (below orange line), studied in detail in a previous study.^13^ Movie S2 shows the 3D rendering of the main elements in sample **30**, and Movie S3 shows the 3D rendering of the main elements in sample **44b**.

**Figure 6:**
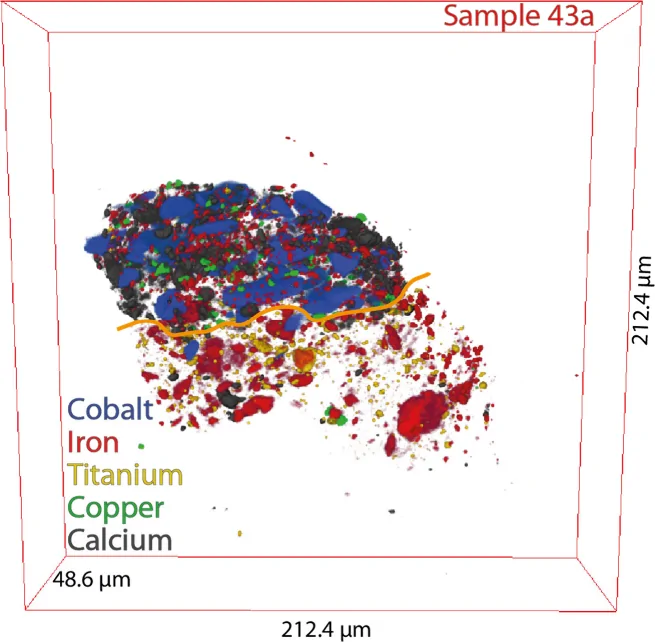
Volume rendering of the main elements detected in sample **43a**. The sample consists of two layers, the ground layer below the orange line and a smalt-containing paint layer on top.

To calculate the volumes of the pigment-related element distribution, the lowest 10% of the intensity range of every element was filtered out, to avoid including the organic fraction which can contain some trace elements. For all three samples, a sub volume of 8 million voxels (64,000 μm^3^) was selected in a representative area of the smalt paint. In Figure S17 the sub volume is shown for sample **44b**. Table 2 shows the volume percentages of the main elements present in the samples: Co, Fe, Ca, and Cu. Sample **44b** contains the highest volume percentage of smalt at 58.6 vol %, followed by sample **30** at 29.1 vol % and then **43a** at 17.2 vol %. Next to the elements and associated pigments mentioned in the text and Table 2, some components of the paint are not detectable by the hard X-ray beamline such as clay-like components (e.g. brown Cassel earth) and the drying oil. The ratio of pigment-to-oil varies among 17th century paints.

**Table 2:** Volume Percentages of the Main Chemical Elements Detected with XRF Tomography from the Three *Night Watch* Paint Samples

	**43a**	**30**	**44b**
cobalt	17.2 vol %	29.1 vol %	58.6 vol %
representative for smalt			

iron	0.5 vol %	0.2 vol %	0.3 vol %
representative for earth pigment			

calcium	10.7 vol % (BB, CC)	10.2 vol % (BB, RYL and CC)	2.1 vol % (RYL, CC)
representative for bone black (BB), red or yellow lake (RYL) or calcite (CC)			

copper	0.5 vol % (AZ or CU)	0.3 vol % (AZ)	2.8 vol % (AZ)
representative for azurite (AZ) or an unidentified copper-containing component (CU)			

All three samples show the presence of copper-containing particles. These particles can be very clearly visualized by using the strong Cu K XRF signal. In Figure 7 the Cu-containing particles are visualized in 3D for all three samples. The shown particles were filtered from the Cu-distribution via automatic thresholding, followed by a watershed segmentation, and subsequently by a separation algorithm to further break down contacting particles. All particles were then assigned a unique label to assess their physical properties. Table 3 shows the mean and median size of the Cu-particles (excluding all clusters with a volume smaller than 100 voxels). More information on the Feret diameters can be found in Table S1. Table 3, together with the 3D rendering in Figure 7, shows that in sample **43a**, the Cu-rich particles are distinctly smaller (median volume of 3 μm^3^) than in the two other samples (46 μm^3^ in sample **30** and 21 μm^3^ in sample **44b**). In sample **44b**, there is a large variety in the volume of the copper particles. In this sample, the presence of azurite (Cu_3_(CO_3_)_2_(OH)_2_) was confirmed by SR-μ-XRD (see Figure S18).

**Figure 7:**
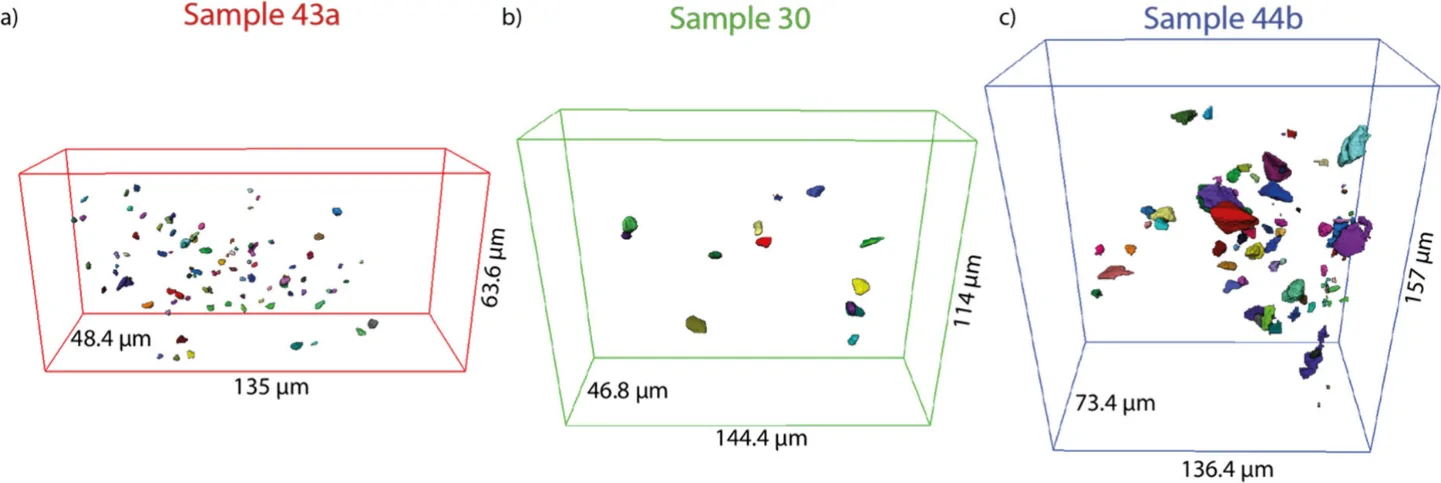
Volume rendering of the segmented copper particles in samples **43a**, **30**, and **44b**.

**Table 3:** Median, Mean, Minimum, and Maximum Copper Particle Volume for the Three Samples^a^

	**43a**	**30**	**44b**
median volume	3 μm^3^	46 μm^3^	21 μm^3^
mean volume	4 μm^3^ (SD 4.2)	48 μm^3^ (SD 30.1)	86 μm^3^ (SD 187.8)

minimum particle volume*	0.8 μm^3^	1.0 μm^3^	0.8 μm^3^
sieved particles below 0.80 μm^3^			

maximum particle volume	30 μm^3^	97 μm^3^	1280 μm^3^
Feret 3D shape (D/d) (median)	1.90	2.02	1.97
number of particles	91	14	83

a All particles consisting of 100 voxels or smaller were excluded.

Azurite is the main copper containing pigment known in Rembrandt’s palette. We assume that in sample **30** the copper-containing particles are also azurite, as this sample was taken in a blue to purple area of the painting. The presence of azurite was also confirmed by short wave infrared reflectance imaging spectroscopy (SWIR-RIS (900–2500 nm)) measurements in the sample location areas of samples **30** and **44b** (see Figure S19). SR-μ-XRD measurements did not detect azurite in sample **43a**, and neither did SWIR-RIS in the sample location area (Figure S19). The median volume of the particles in sample **43a** (taken in the dark background) is 10–16 times smaller than the median values for samples **30** and **44b**. This leads to the hypothesis that either a different copper-containing component was used here, or very small azurite particles were added for a different purpose than its bright blue color. Some literature describes that Rembrandt used azurite in dark background or shadow areas.^2,9^ Unfortunately, there were also no positive matches with other copper-containing pigments with SR-μ-XRD and Raman spectroscopy in sample **43a**. The copper-containing component might also be a verdigris (collective term for different copper­(II) acetates), which is suggested by multiple historic sources to have been added to black paint. One of the reasons to add this would be to increase the drying properties of the paint.^57,58^

Calcium (see Figure 6) was detected in sample **43a**. In this dark paint the calcium is related to the use of bone black, which was also identified by LM and SEM-EDX by the presence of both Ca and P (see Figure S20). Calcium is also found in the other two samples. Sample **30** contains some bone black; however, an additional source of calcium in this sample may be related to yellow and red lakes. Calcium, mostly in the form of calcium carbonate (CaCO_3_), is added as a mordant to an organic dye to create an insoluble colored complex that results in a lake pigment.^8,59^ Since in sample **44b** we did not detect any bone black with LM or SEM-EDX, we suspect the calcium to be related to red and yellow lakes. Some calcium-containing particles might also be pure grains of calcite (CaCO_3_).

Iron is also present in all three samples as small particles throughout the paint layer. The presence of iron often indicates the use of an earth pigment. Figure 6 shows that the ground layer in sample **43a** also contains a lot of iron-bearing particles, agreeing with the previous XRF tomography study on the ground of *The Night Watch*.^13^

Mercury is found in all studied paint samples as well. In oil paintings, the presence of mercury is generally associated with the pigment vermilion (HgS). In all three samples, the mercury particles are very small spheres, except for two larger particles in sample **44b**. These small particles can be easily overseen when using SEM-EDX mapping, especially if an overview measurement of the sample is taken instead of detailed maps of a specific paint layer. This shows again the added value of SR-μ-XRF tomography to provide a more complete view of the composition of the paint in comparison to standard 2D techniques. Figure S21 shows an example EDX spectrum collected in one of the vermilion particles in sample **44b**.

Lead is also present in all samples. Lead is present as clearly distinguishable particles in the paint layer as well as featuring a more homogenous signal. In the case of particles, we assume either some lead white or lead tin yellow (lead stannate, Pb_2_SnO_4_) was added to the paint. Some Sn was detected by μ-XRF in samples **43a** and **44b**. The more homogenous signal in sample **30** is present in the small section of the sample that is part of the ground layer. The elevated level of lead here is associated with dispersed lead originating from the lead-containing impregnation “layer” of *The Night Watch*.^13^

## Discussion

In this study we have shown the potential of micro-X-ray fluorescence tomography to study individual smalt particles and to determine the composition of smalt-containing paint mixtures in Rembrandt’s paints from his masterpiece, *The Night Watch*. Smalt pigment particles were segmented based on the 3D cobalt distribution, and the median particle size was calculated for the different samples. Sample **30** has the smallest particles (∼62 μm^3^), followed by sample **43a** (∼98 μm^3^), and the particles in sample **44b** were considerably larger (∼186 μm^3^). Based on the quantification of the volume percentage of the smalt particles in the three paint samples, we see a large variation in the smalt content (17, 30, and 60 volume percent, corresponding to samples **43a**, **30**, and **44b**, respectively).

It is likely the differences in size and proportion point towards deliberate artistic decisions to use different types or grades of smalts for different purposes. In sample **43a**, the smalt is used in a dark brown background paint. This sample has the lowest volume percentage of smalt. In this case, the smalt might have been added not for chromatic reasons primarily, but instead to improve the paint’s drying properties, or to make the paint thicker.^8^ The particle size of the copper-containing particles in sample **43a** are much smaller than the Co-containing particles in samples **30** and **44b**. It appears that Rembrandt used azurite or another Cu-compound for different purposes in this dark background compared to the figurative areas where the Cu-containing particles are much larger.

Sample **30** is from a paint that probably had a dark purple/blue color. Next to ∼30 vol % of smalt, bone black was added to darken the paint. The sample is taken from the shadow area of the clothing of the shooting musketeer. We expect that in this paint, smalt has been added primarily for its blue color.^39^ Azurite and some earth pigments were also added to the paint mixture. Next to the bone black, some of the calcium present in this paint mixture probably originated from red or yellow lakes, or calcite. It is necessary to determine the exact species with additional techniques such as μ-XRD or Raman spectroscopy, as the element specific information obtained by XRF is insufficient to distinguish between species containing the same chemical elements.

Sample **44b** must have come from the (originally) most colorful area, as it mainly consists of smalt, azurite, and red lake. This sample has both the highest volume percentage and the largest particles of smalt. In this area, the smalt paint was used for the upper layer employed to paint the colored feathers on Rombout Kemp’s hat. Rembrandt is known for his use of *impasto* paints, i.e., paint with a three-dimensional structure that is applied thickly on the surface. He might have therefore intentionally used a coarser smalt in this area, to emphasize the feathers and to influence the reflection of light on these feathers. Considering the high proportion of smalt in the paint layer, it is likely that besides its effect on the physical properties of the paint, the smalt was also used for its blue color. Sample **44b** also contains the highest volume percentage of copper, adding to the hypothesis that Rembrandt intended to use a blue to purple paint here. The red component of this paint would come from added-in earth pigments, some vermilion and red lake particles.

There is a high level of heterogeneity between smalt particles within every sample, in both size and elemental fingerprint. Although the use of different types of smalt between the three samples was expected, this study shows that the variance within the samples is far larger than previously expected. The concentration of cobalt varies considerably among particles. The clustering results show that the variety in concentration of bismuth is one of the main factors in the clustering process. This heterogeneity can either originate from the use of a mixture of different smalt types for one paint, heterogeneity in the cobalt ore, differences caused during the smalt production process, or possibly due to *in-situ* degradation of the pigment. If the heterogeneity within the smalt paints is due to the mixing of several smalt types for one paint, we are unable to answer whether these were mixed on purpose by Rembrandt or that the smalt types were already mixed when the smalt was bought by him. It seems most likely that the large variety in Co–Bi ratio was already in the smalt powder(s) used by Rembrandt. Glass may be a very heterogenous material and the procedure of making smalt likely was not performed in an extremely controlled manner in the 17th century. A quantitative comparison of the elemental fingerprint among the samples could not be performed due to absorption effects in the tomographic XRF results. The large variety between smalt particles within the paint samples emphasizes that the heterogeneity of pigments may be larger than expected. This should be taken into consideration when studying historical paint samples. To distinguish smalt types (or other pigments), enough data points should be collected, either on many samples or by using more bulk-type analytical techniques such as MA-XRF, although due to absorption effects, these results will remain semi-quantitative. Microscale studies are very valuable to characterize individual particles and degradation phenomena.

## Conclusion

In conclusion, considerable variety was encountered in the size and composition of the smalt particles used by Rembrandt in *The Night Watch*. The high sensitivity and spatial resolved information from SR-μ-XRF tomography enabled us to semi-quantify these differences. This semi-quantitative information (in volume percentages) gained on the composition of the paint mixtures used by Rembrandt, brings us closer to the creative process of the Old Master, and can be used to improve paint model systems and reconstructions for further research on the intention of the artist, ongoing degradation phenomena and the optimal treatment methods for these paints.

Our results point toward the use of different types of smalt paint for the three areas of *The Night Watch* studied here, based on particle size, elemental fingerprint, and addition of other pigments. The different smalt-containing paint mixtures emphasize that Rembrandt carefully prepared his pigment mixtures for each area and also took into account the effects of the physical properties of the paint. Within the samples, the heterogeneity among smalt particles was far larger than expected; it appears that multiple types of smalt were used by Rembrandt to make one paint mixture. Other explanations could be that the high heterogeneity of the glass pigment smalt is due to either the source or preparation of the material or due to degradation of the pigment. The high variety within the samples stresses the importance of investigating a large number of smalt-containing samples or smalt particles before drawing conclusions on the use of one or multiple types of smalt.

Ultimately, this study highlights the power of SR-μ-XRF tomography as a highly sensitive tool for advanced chemical analysis, demonstrating its ability to semi-quantify the composition of complex, heterogeneous matrices. Beyond art conservation science, the analytical framework presented here represents a significant contribution to the broader field of analytical chemistry. The ability to non-destructively isolate, chemically fingerprint, and volumetrically cluster micro-particles within a dense matrix offers immense potential for the analysis of other complex materials systems, ranging from advanced functional glasses and heterogeneous catalysts to geological samples and engineered composite materials.

## Supplementary Material









## Data Availability

All data are available in the main text or the supplementary materials.

## Notes

Additional data analysis details, materials, and methods, including SEM-EDX tables on the composition of smalt particles and 3D renderings of the elements in the studied samples­(PDF)
3D rendering of sample **43a**, with the distribution of Ca (K) in black, Co (K) in blue, Cu (K) in green, Fe (K) in red, K (K) in cyan and Ti (K) in yellow (MP4)
3D rendering of sample **30**, with the distribution of Ca (K) in black, Co (K) in blue, Cu (K) in green, Fe (K) in red, K (K) in cyan and Ti (K) in yellow (MP4)
3D rendering of sample **44b**, with the distribution of Ca (K) in black, Co (K) in blue, Cu (K) in green, Fe (K) in red and K (K) in cyan (MP4)
