# Supplementary material for: Visualizing and Quantifying Rembrandt’s Smalt Mixtures in The Night Watch in Three Dimensions Using μ‑X-ray Fluorescence Tomography
Source: Anal Chem. 2026 Sep 17;98(38):27858–72. doi: 10.1021/acs.analchem.5c07178 (PMC13629168; doi:10.1021/acs.analchem.5c07178)
Supplement: Supplementary file 1 [file ac5c07178_si_001.pdf]

Supporting information

Visualizing and quantifying Rembrandt's smalt mixtures in *The Night Watch* in three dimensions using µ-X-ray fluorescence tomography

Fréderique T. H. Broers,[1,2,3,4]* Annelies van Loon,[2] Jan Garrevoet,[5] Francesca Gabrieli,[2] Jorien R. Duivenvoorden,[2] Victor Gonzalez,[2,6] Paul J.C. van Laar,[7,8] Petria Noble,[2] Koen Janssens,[3] Florian Meirer,[4] Katrien Keune[1,2]

Affiliations

[1] Van't Hoff Institute for Molecular Sciences, University of Amsterdam, Science Park 904, 1090 GD Amsterdam, The Netherlands.

[2] Science Department, Conservation & Science, Rijksmuseum, Hobbemastraat 22, 1071 ZC Amsterdam, The Netherlands.

[3] Antwerp X-ray Imaging and Spectroscopy Laboratory, University of Antwerp, Groenenborgerlaan 171, 2020 Antwerp, Belgium.

[4] Inorganic Chemistry & Catalysis, Institute for Sustainable and Circular Chemistry, Utrecht University, Universiteitsweg 99, 3584 CG Utrecht, The Netherlands.

[5] Photon Science at Deutsches Elektronen-Synchrotron DESY, Hamburg, 22607, Germany

[6] Université Paris-Saclay, ENS Paris-Saclay, CNRS, PPSM, 4 Av. des Sciences, 91190 Gif-sur-Yvette, France.

[7] Hamilton Kerr Institute and Conservation and Science Division, Fitzwilliam Museum, University of Cambridge, Trumpington Street, Cambridge, CB2 1RB, United Kingdom

[8] Department of Conservation & Restoration, VICARTE, NOVA Universidade de Lisboa, 2829-516 Caparica, Portugal

Abstract

Smalt is one of the main blue pigments in Rembrandt van Rijn's *The Night Watch* (1642). Smalt is a cobalt-containing potash glass, ground to a powder to be used as an artists' pigment. In this study, three smalt-containing paint samples from this painting were studied using synchrotron radiation-based micro-X-ray fluorescence (SR-µ-XRF) tomography. To study the original intentions of the artist, the degradation phenomena taking place in the paint and the best preservation methods for the painting, a fuller understanding of the smalt-containing paint mixtures is desired. The smalt particles in Rembrandt's paints show a wide variety in size and elemental composition. Elements related to smalt are e.g. cobalt, potassium, arsenic, bismuth, nickel and iron. The heterogeneity is very high among the particles within one paint sample. Different clusters of particles can be established based on the intensity of bismuth. This variety could be caused by heterogeneity in the cobalt ore, the glass-making process, the use of multiple types of smalt or it might be the result of *in-situ* degradation. Next to the smalt, the volume percentages taken up by other components of the paint are semi quantitively estimated based on elemental information. This information can be used in follow-up studies to improve the accuracy of models and the representativeness of reconstructed paint mockup samples. SR-µ-XRF tomography proves to be a valuable tool for the identification and quantification of complex heterogenous smalt paints.

Table of contents

Segmentation procedure in Avizo S2
Particle size information S3
Element distribution in 3D S6
Clustering method S7
Scanning electron microscopy–energy dispersive X-ray analysis (SEM–EDX) S12
Correlation cobalt and potassium S13
Additional figures S15
Estimation of spatial resolution of the 3D reconstructions S18

Segmentation procedure in Avizo

Settings used in Avizo for the segmentation procedure in Avizo

Median filter

Interpretation	3D
Neighborhood	6 (26 results in too much smoothing)
Iterations	3
Type	Iterative

H-Extrema Watershed

Interpretation	3D
Neighborhood	26
Object Type	White
Marker extent	2000 (044) 3000 (043, 2000 split up particles)
Output type	Split
Algorithm mode	Repeatable

Interactive thresholding

Intensity range 10% of the lowest value (044 → 1638 – 16219) max 163831
(043 → 1638 – 16219) max 163831

Labeling

Interpretation 3D
Neighborhood 6

Label Analysis

Sieve Analysis

Sieve below 100

030

This sample contained one very intense particle that complicated the thresholding and segmentation. Therefore, this particle was filtered out, although some of the edges of the particle could not be easily removed.

The following procedure was executed for sample 30:

>Median Filter
>Interactive Threshold to select high intensity particle 0.166375-2.22846
>Invert
>Mask
> H-extrema Watershed, Marker extent 2000 (see above)
> **Auto thresholding**

Auto Segment 3 Phases
3D
min-max
factorization

Interactive thresholding

Only '2'

Separate objects

Method	Chamfer
Interpretation	3D
Neighborhood	6
Marker extent	4
Output type	Split
Algorithm mode	repeatable

Labeling

Interpretation 3D
Neighborhood 6

Label Analysis

Sieve Analysis

Sieve below 100

Labeling

Interpretation 3D

Label Analysis

Segmentation copper

Auto Threshold High

Type	Auto Threshold High
Interpretation	3D
Mode	min-max
Criterion	factorisation

Separate objects

Method	Chamfer
Interpretation	3D
Neighborhood	26
Marker extent	4
Output type	Split
Algorithm mode	repeatable

Particle size information

Table S1: Additional information on the Feret diameters calculated in Avizo.

	43a	30	44b
**Cobalt** Median of the maximum of the Feret diameters (Length3D in Avizo)	10.2 μm	8.7 μm	12.4 μm
**Cobalt** Median of the minimum of the Feret diameters (Width3D in Avizo)	4.4 μm	4.1 μm	6.3 μm
**Copper** Median of the maximum of the Feret diameters (Length3D in Avizo)	2.9 μm	7.3 μm	6.0 μm
**Copper** Median of the minimum of the Feret diameters (Width3D in Avizo)	1.5 μm	3.3 μm	3.1 μm

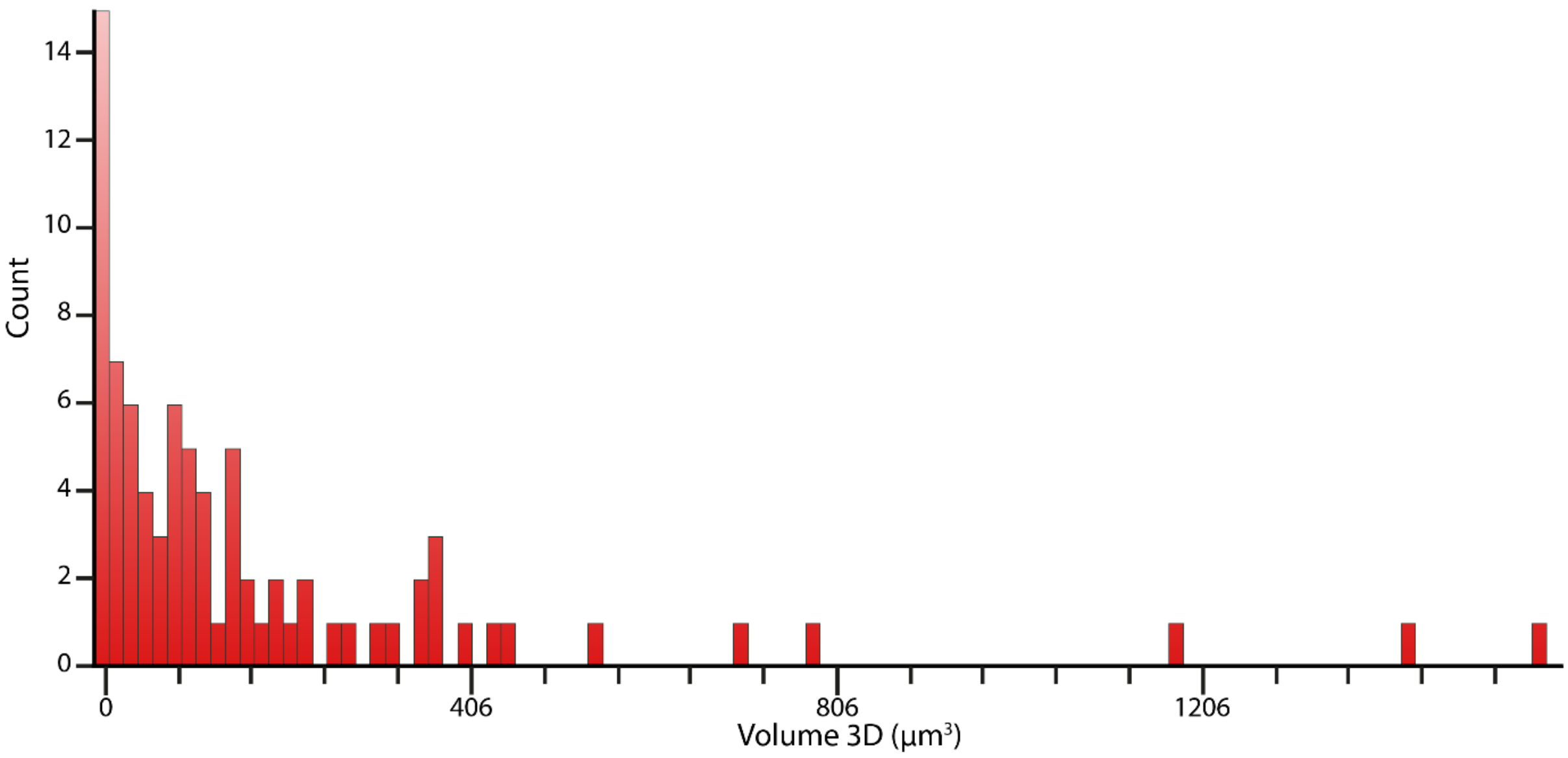

Figure S1: Particle volume distribution of smalt particles in sample 43a.

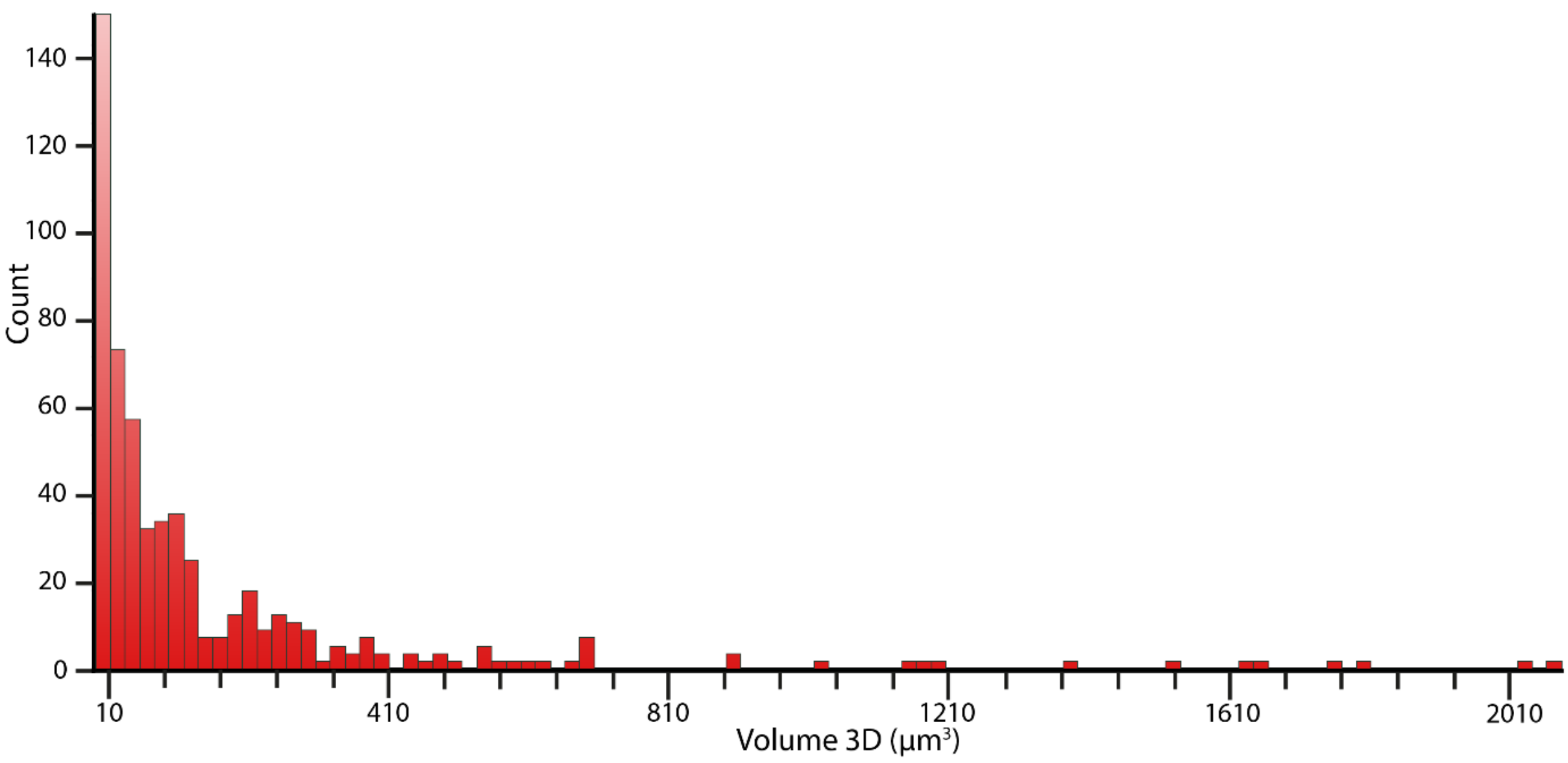

Figure S2: Particle volume distribution of smalt particles in sample 30.

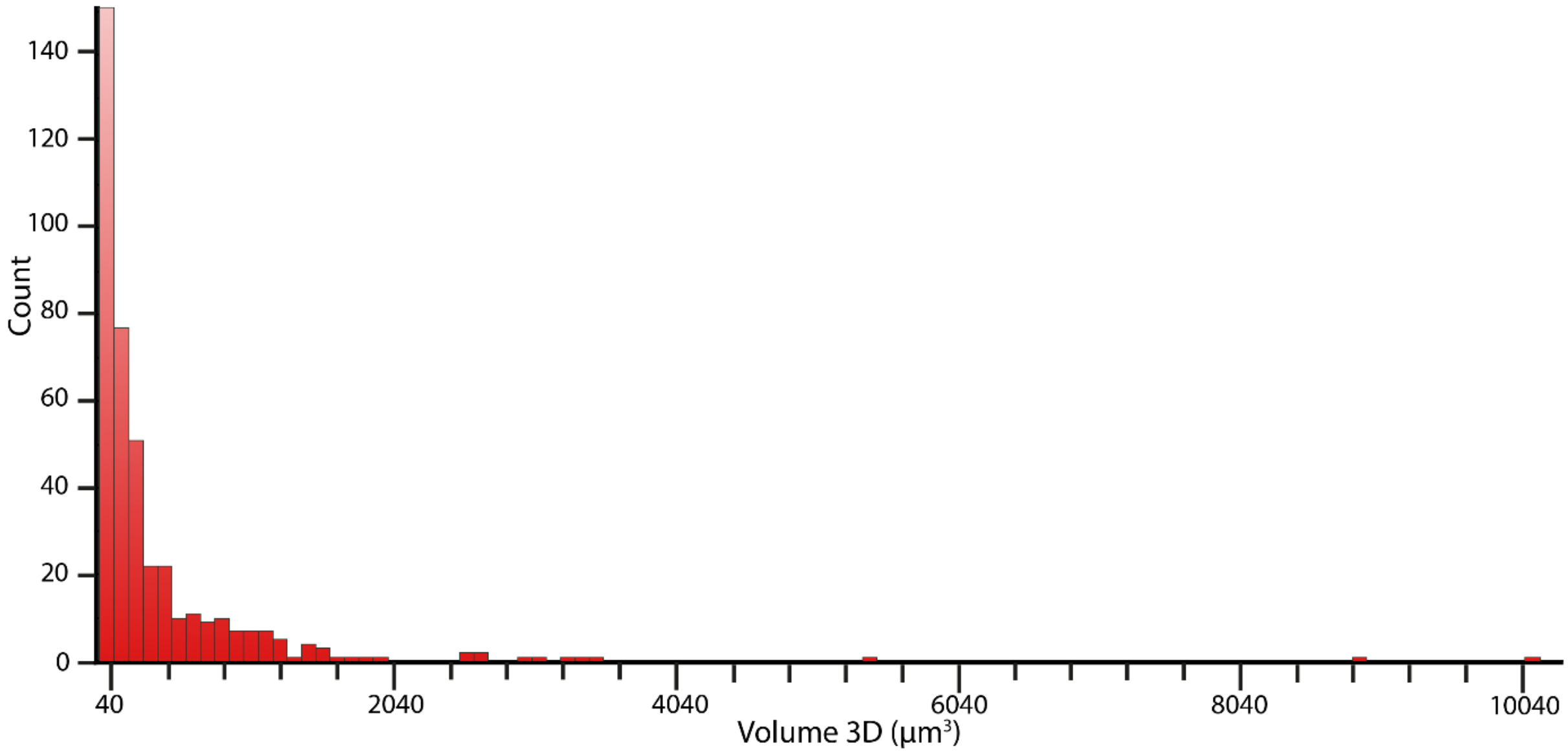

Figure S3: Particle volume distribution of smalt particles in sample 44b.

Element distribution in 3D

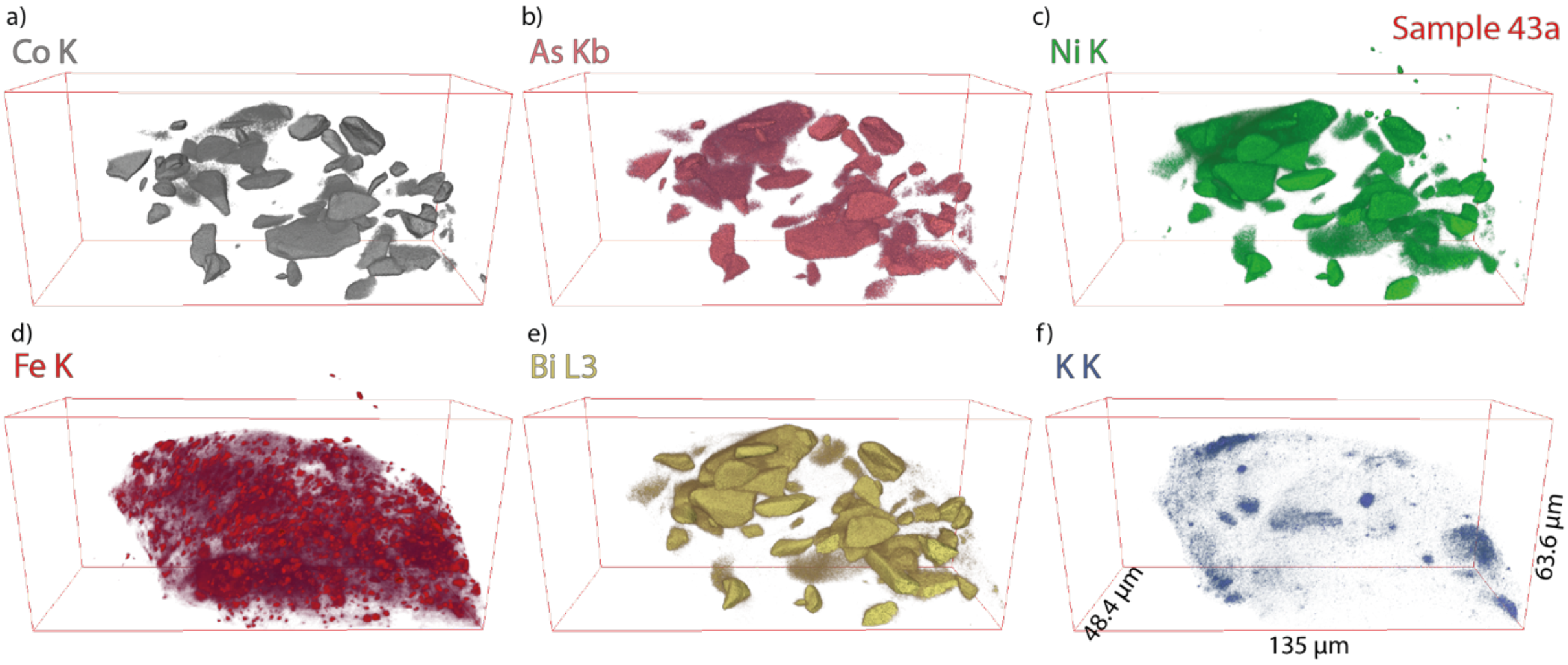

Figure S4: Elemental distribution in 3D of (a) cobalt, (b) arsenic, (c) nickel, (d) iron, (e) bismuth and (f) potassium in sample 43.

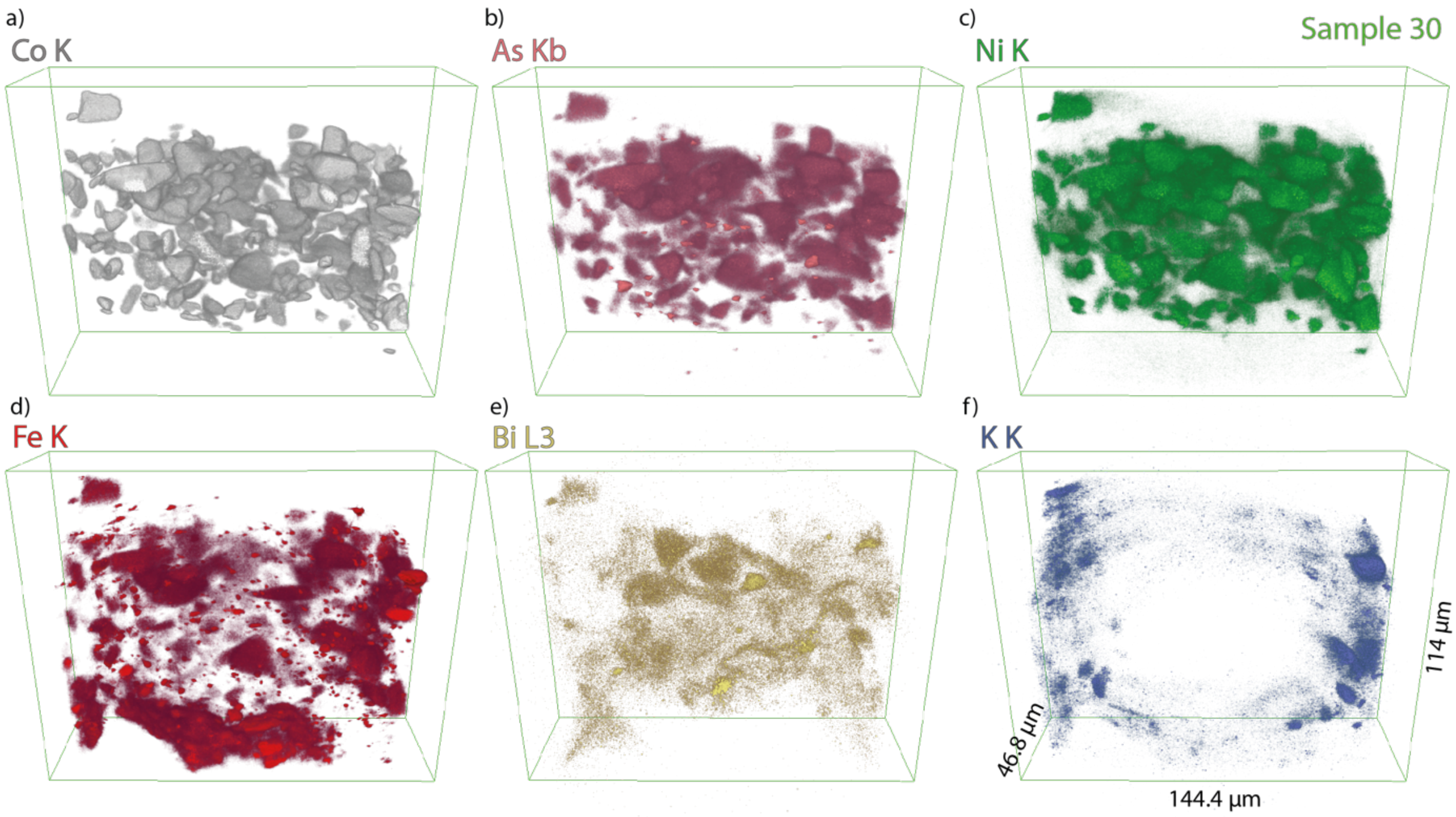

Figure S5: Elemental distribution in 3D of (a) cobalt, (b) arsenic, (c) nickel, (d) iron, (e) bismuth (f) and potassium in sample 30. The small particle around the smalt in (b) are related to the presence of vermillion (HgS), due to overlap op the As-Kb fluorescence lines with Hg-Lb fluorescence lines.

Clustering method

An in-house MATLAB script was written to perform cluster analysis of the XRF intensities of the elements in the smalt particles. A k-means clustering and an agglomerative hierarchical cluster tree both showed very similar results, and it was decided to continue with the results of the agglomerative hierarchical cluster tree. Where possible, the number of clusters were defined by the Davis Bouldin Value (DBV). The lowest DBV value, corresponds the most to the hypothetical 'ideal' clustering.[56] However, the DBV value is sensitive to outliers, and in a few cases such as in the example given below, the value suggested by DBV resulted in insufficient clusters due to outliers with very high values in one or multiple elements. In this case the next minimum in the DBV plot was chosen. Note: The colors in the tree do not match the colors in the cluster plot.

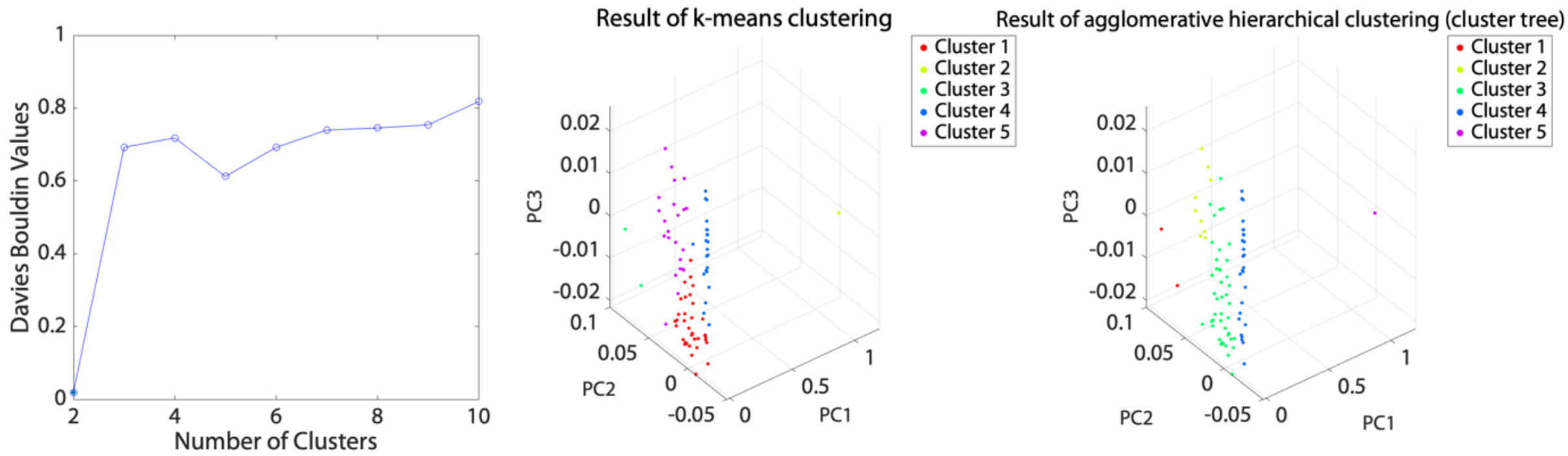

Figure S6: Clustering result for sample 43a. Five clusters were selected, as the DBV of 2 only separated the outlier on the right (yellow in the k-means and magenta in the cluster tree) from the other particles.

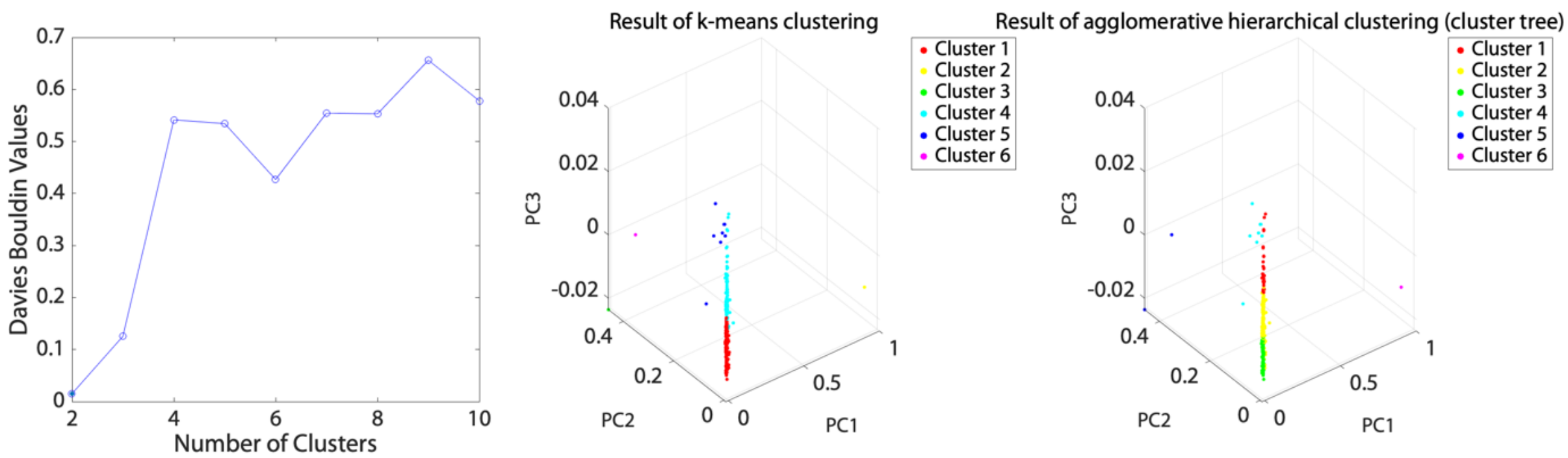

Figure S7: Clustering result for sample 30. Six clusters were selected, as the DBV of 2 or 3 only separated the outliers from the other particles.

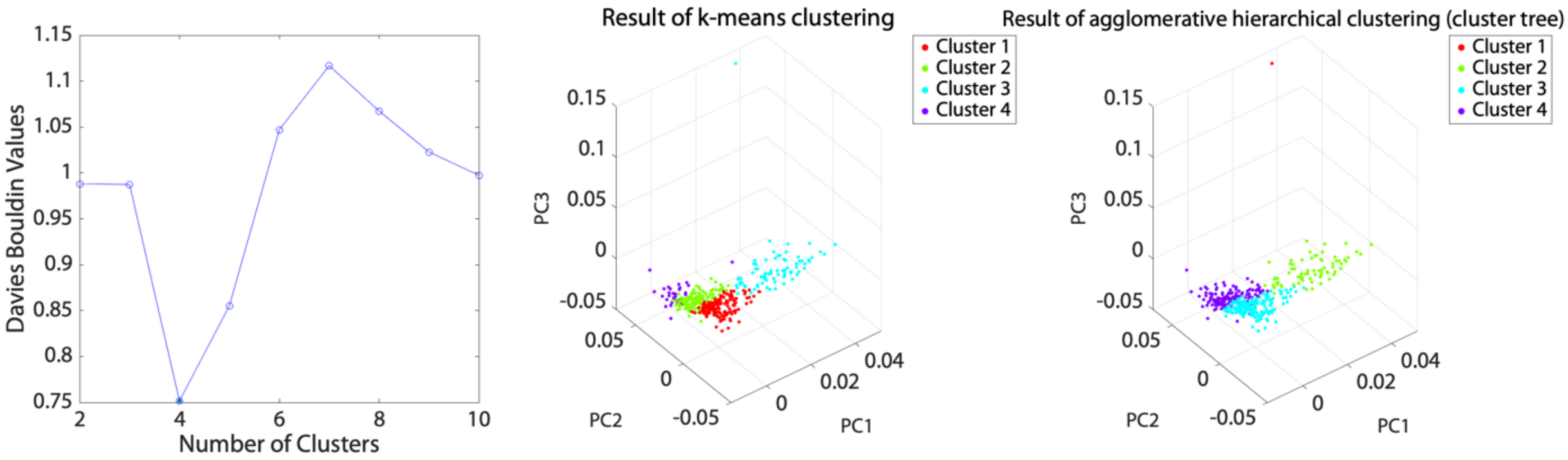

Figure S8: Clustering result for sample 44b. Four clusters were selected as suggested by the DBV. There is one outlier (red cluster on the right), that is well-separated from the other particles by the agglomerative hierarchical clustering.

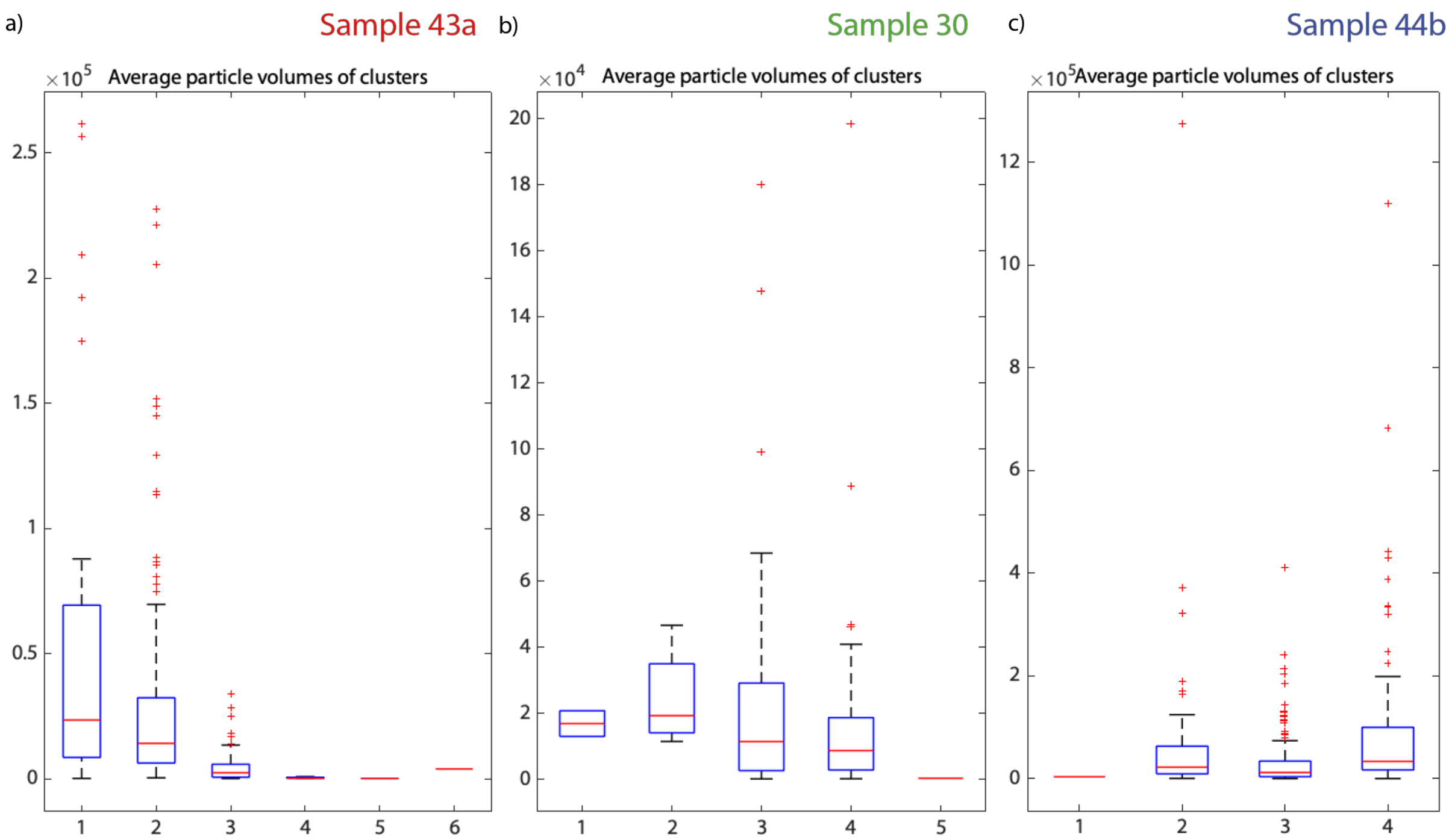

Figure S9: Average particle volume of the different clusters of samples (a) 43a, (b) 30 and (c) 44b.

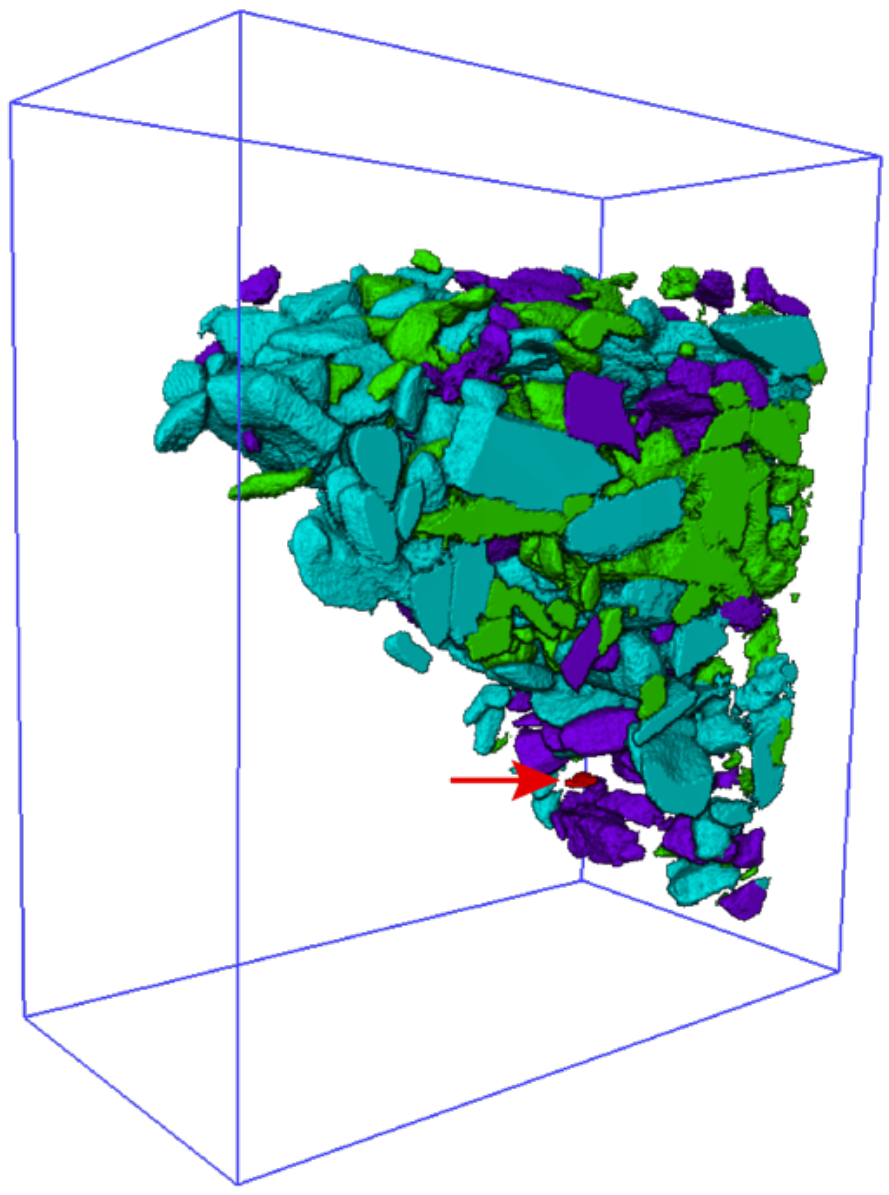

Figure S10: Clustered 3D rendering of sample 44b, shown in a different orientation to show the red particle (middle of the bottom area of the sample).

a) Sample 43a

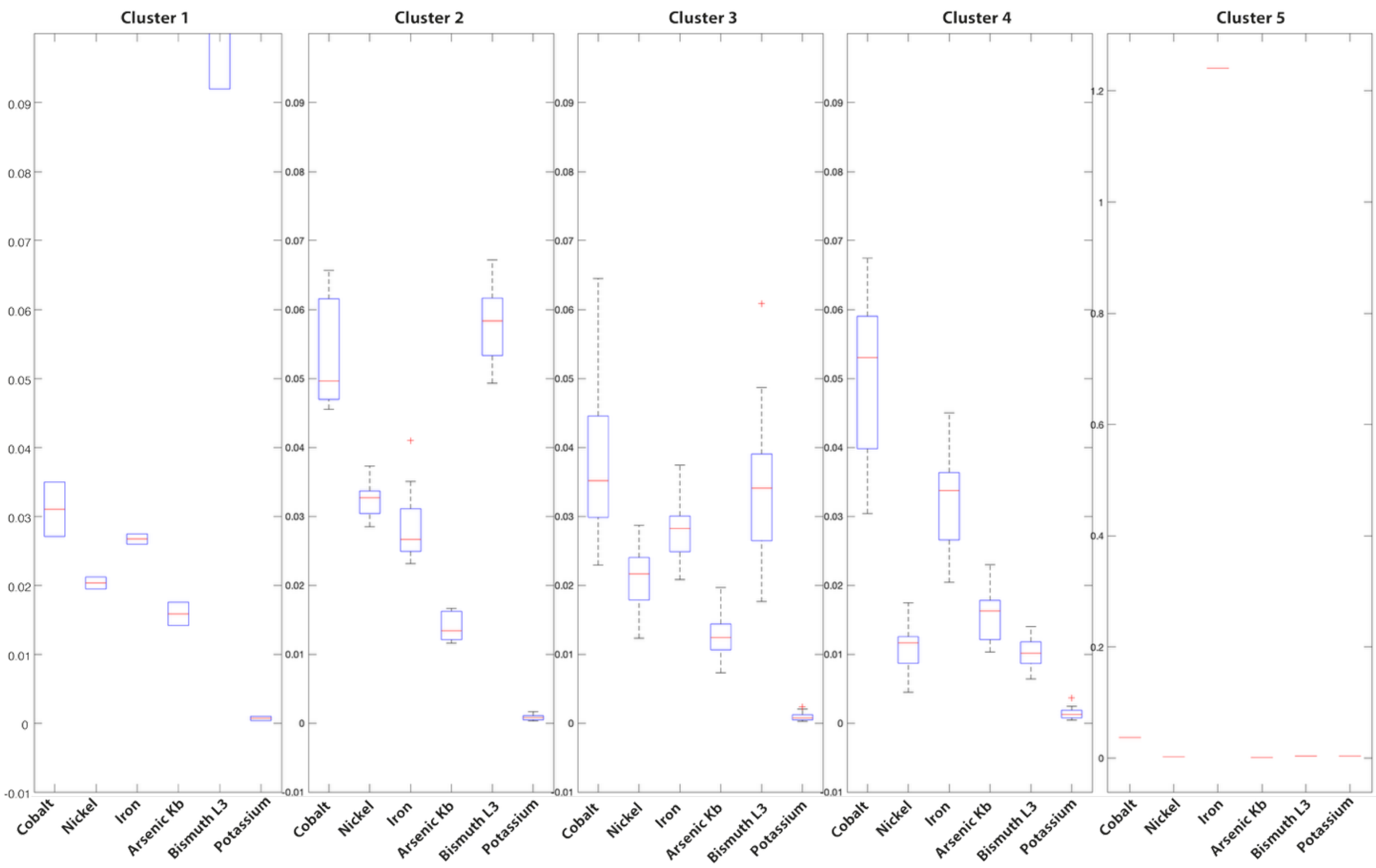

b) Sample 30

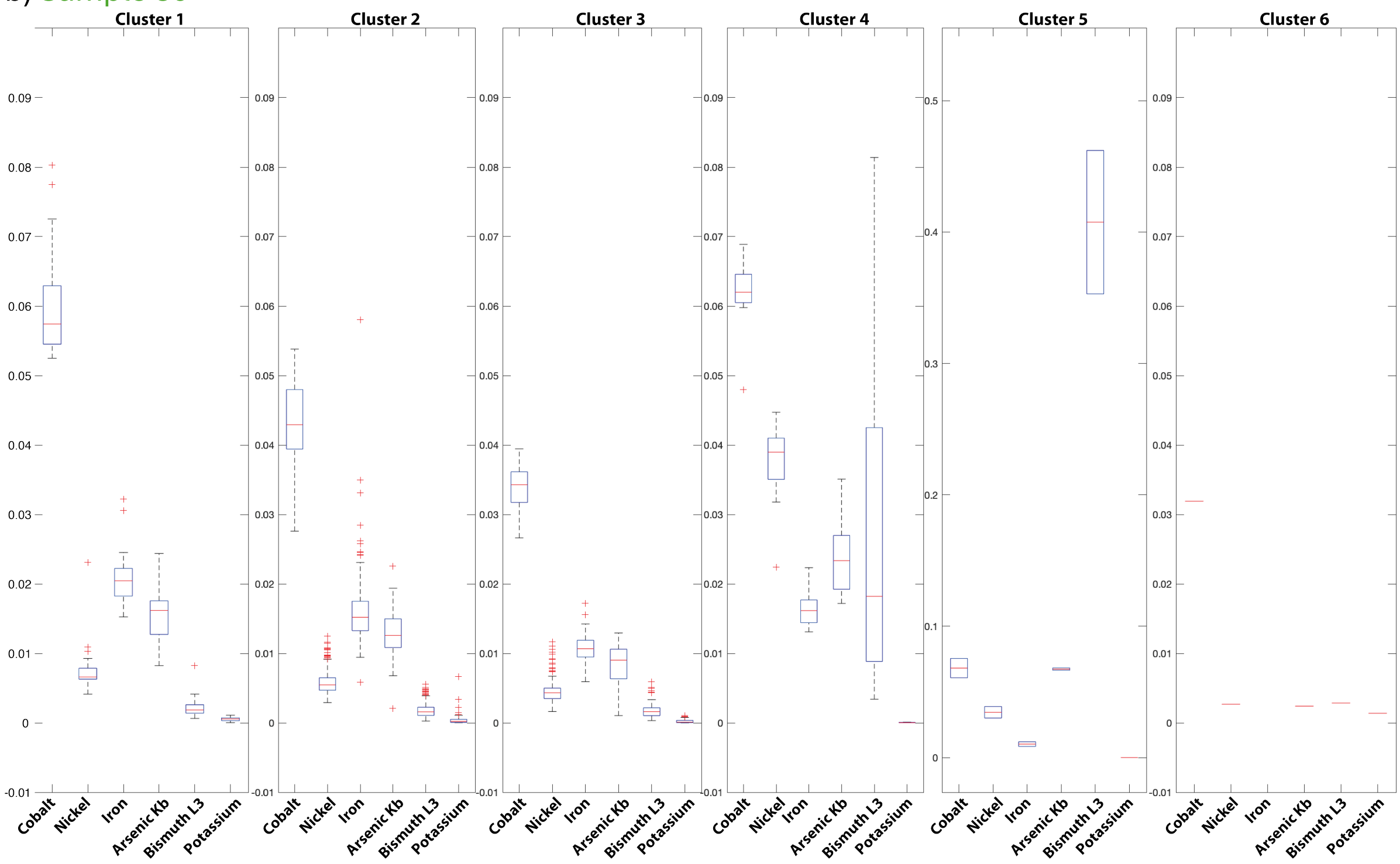

c) Sample 44b

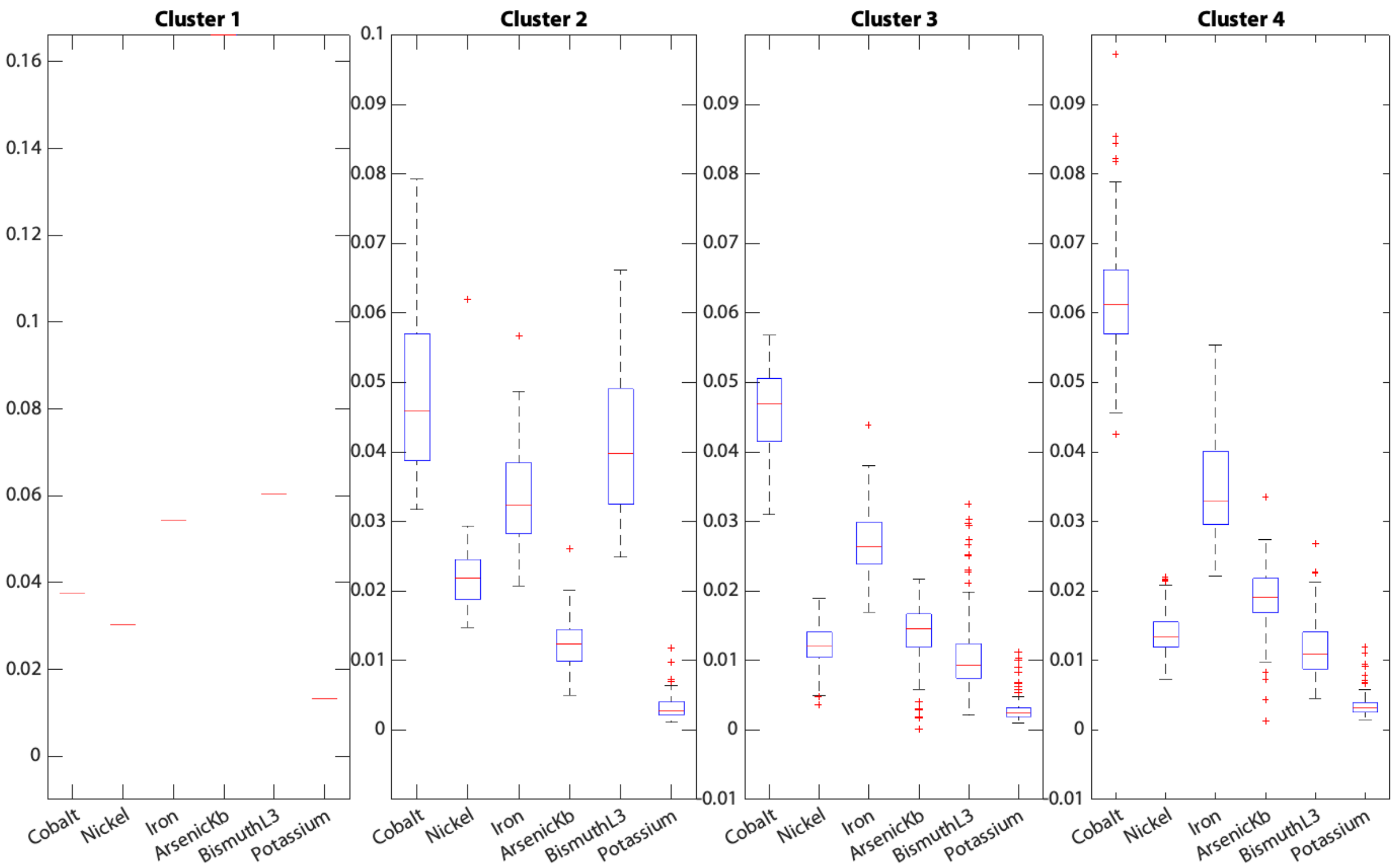

Figure S11: Variation and average intensity per element for the different clusters of sample a) 43a, b) 30 and c) 44b. Note that clusters showing only one stripe consist of one particle with an extreme value of one of the elements.

a) Sample 43a

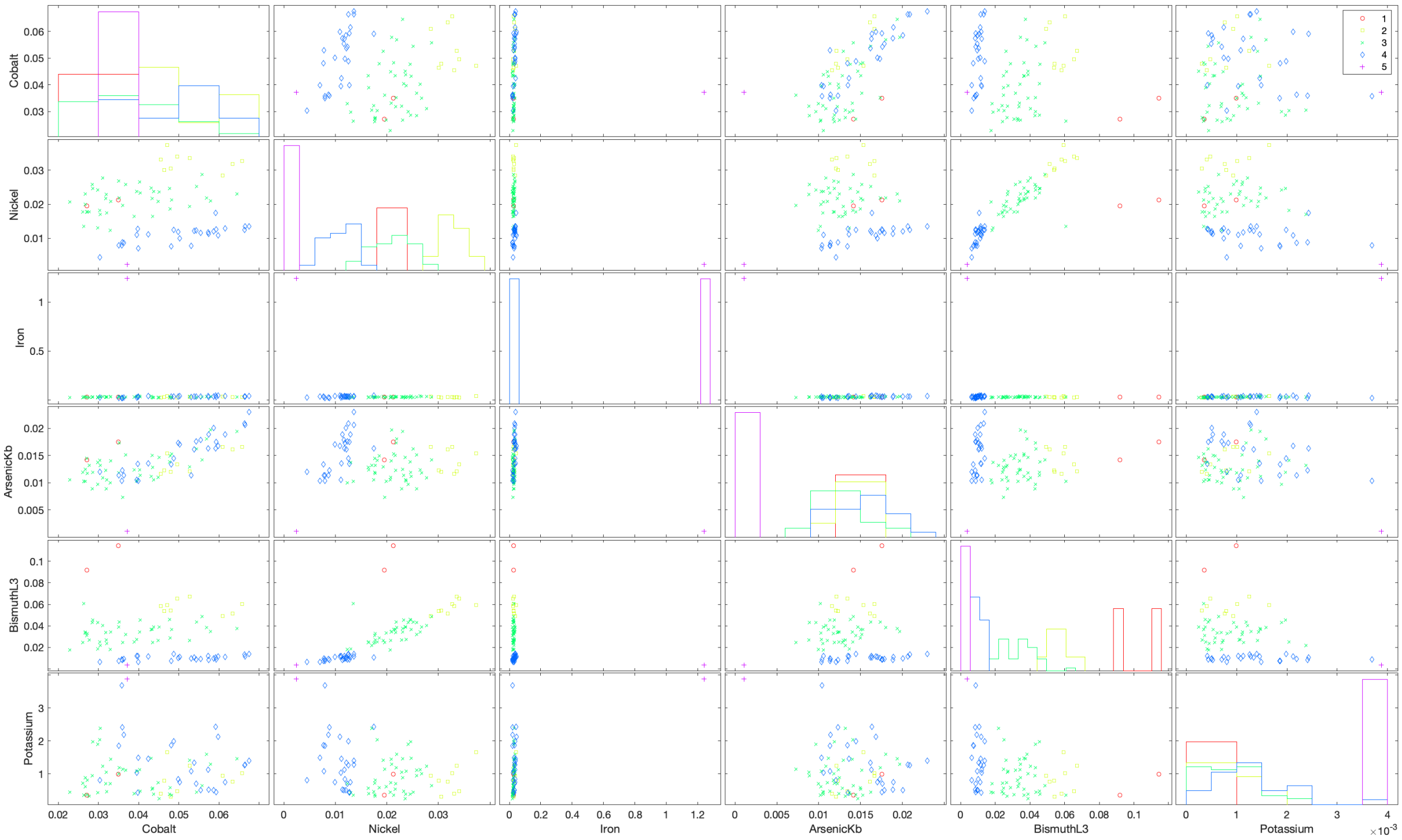

b) Sample 30

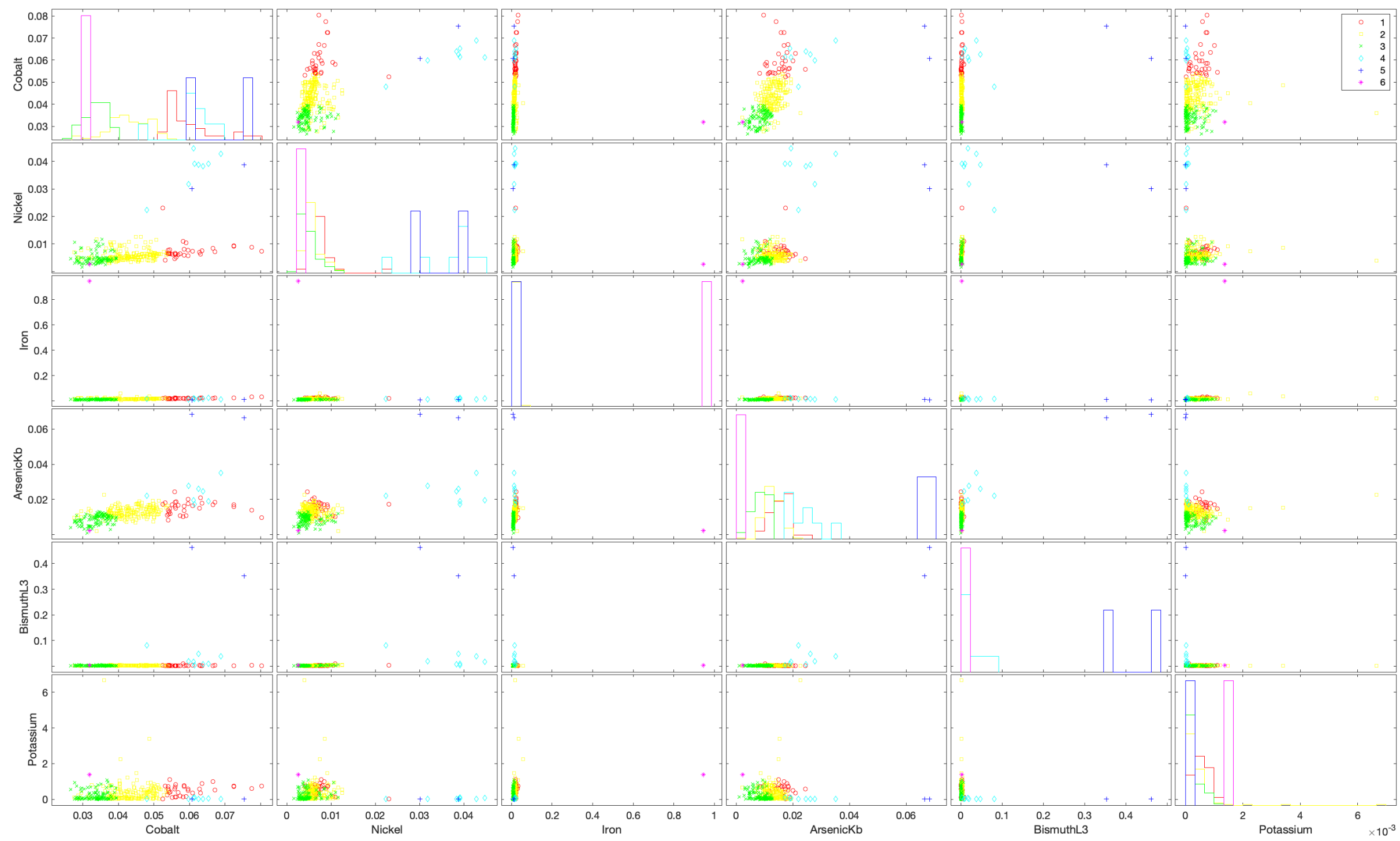

c) Sample 44b

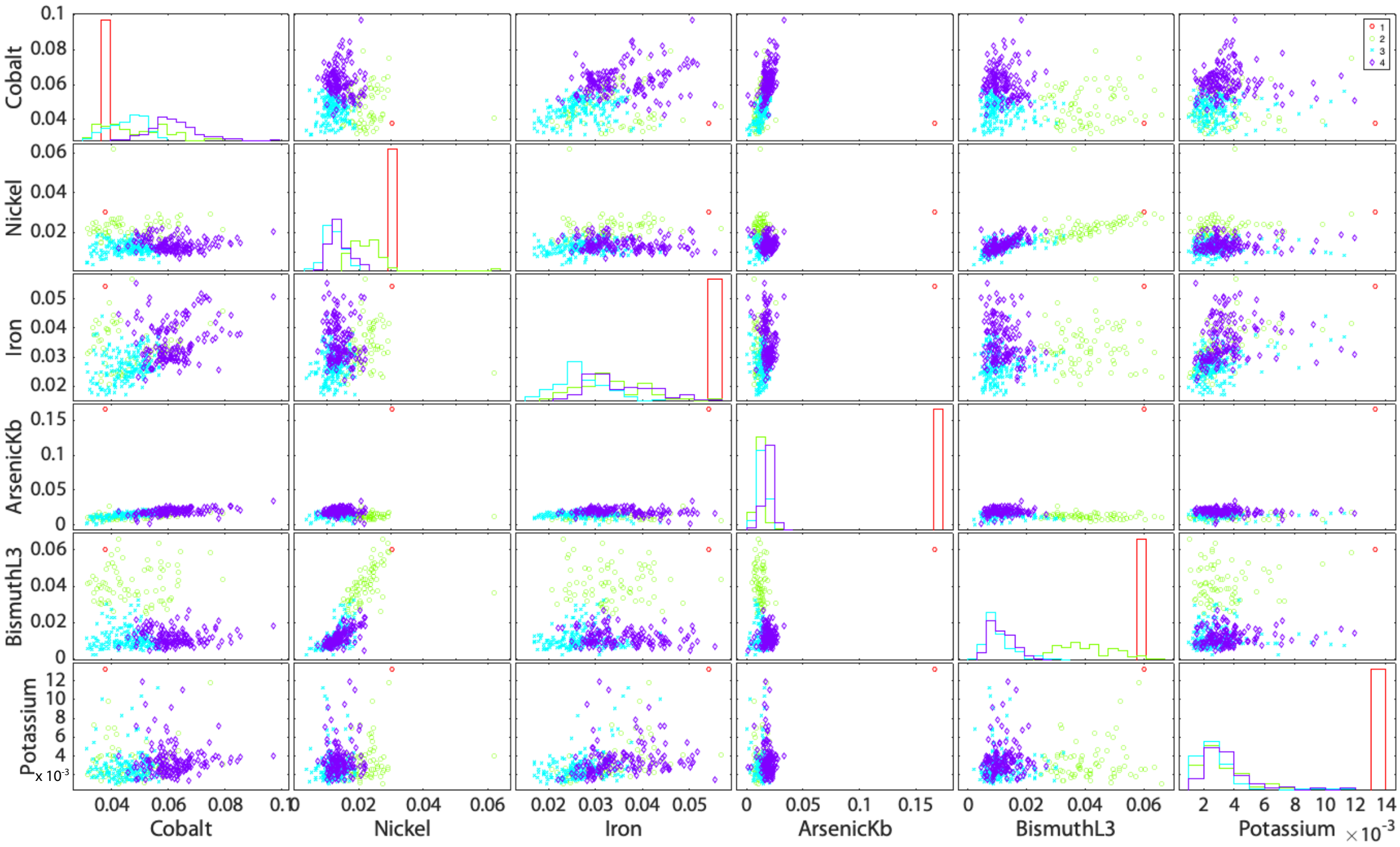

Figure S12: All correlation plots of the elements, for sample a) 43a, b) 30 and c) 44b.

Scanning electron microscopy–energy dispersive X-ray analysis (SEM–EDX)

SEM-EDX data were collected on smalt particles in samples from the same area (43 and 44) or in the analyzed sample (30). High-lighted values are deviating 1 standard deviation above (yellow) or below (red) the average value of the smalt particles in that sample. The number of measurements per sample differ due to the number of smalt particles available in the cross section surface. Smalt particles with a high potassium level are outlined in black (well-preserved).

Table S2: Chemical composition of eight smalt particles in sample SK-C-5_043. Values in yellow are deviating more than one standard deviation above the average and values in red are more than one standard deviation below the average. The well-preserved smalt particle is outlined with a black line.

**SK-C-5_043**	**1**	**2**	**3**	**4**	**5**	**6**	**7**	**8**	**Average degraded**	SD
Na_2O	-	0.4	-	-	-	-	0.1	-	**0.1**	0.15
MgO	0.9	0.9	0.6	0.9	0.5	0.5	0.7	1.3	**0.8**	0.28
Al_2O_3	1.4	2.4	1.5	2.5	2	2.4	1.7	2	**2.1**	0.41
SiO_2	82.6	80.8	76.4	85.9	86.2	83.6	83.4	78.5	**83.0**	2.72
K_2O	2.6	0.4	7	1.1	0.8	4.7	1.1	5.7	**2.3**	2.09
CaO	1.1	2.2	0.7	2.7	0.7	1.3	0.7	1.7	**1.5**	0.76
Fe_2O_3	2.1	4.3	4.4	3.1	4.1	3.3	3	3.6	**3.4**	0.74
CoO	3.5	3.7	4.4	0.9	1.6	1.1	3.6	3.4	**2.5**	1.28
NiO	0.6	0.5	0.2	0.1	0.4	0.3	0.7	0.2	**0.4**	0.22
As_2O_3	3.9	4.4	4.9	2.7	2.9	2	2.7	2.9	**3.1**	0.81
Bi_2O_3	1.3	-	-	-	0.9	0.7	2.3	0.4	**0.8**	0.81
	100	100	100	100	100	100	100	100		

Table S3: Chemical composition of eighteen smalt particles in sample SK-C-5_030. Values in yellow are deviating more than one standard deviation above the average and values in red are more than one standard deviation below the average. The well-preserved smalt particles are outlined with a black line.

SK-C-5_030	1	2	3	4	5	6	7	8	9	10	11	12	13	14	15	16	17	18	Average well-preserved	SD	Average degraded	SD
Na_2O	0.2	0.7	0.2	-	0.5	0.4	0.2	0.2	0.8	0.4	0.5	0.5	0.5	0.6	-	0.4	0.1	0.3	**0.5**	0.24	**0.3**	0.20
MgO	0.3	0.4	0.4	0.2	0.5	0.1	0.1	0.5	0.7	0.2	0.2	-	-	0.5	-	0.3	0.2	-	**0.4**	0.17	**0.2**	0.18
Al_2O_3	2.2	2.7	1.6	2.1	2.2	1.8	2.5	3	2.1	1.9	1.9	1.9	1.7	2.3	1.7	2.3	2	2.1	**2.3**	0.23	**1.9**	0.65
SiO_2	65.4	65	78.9	78.1	76.3	80	79.8	76.1	66.6	78.7	77.6	78	78.7	62.7	79.6	60.8	77.8	77.3	**64.1**	2.32	**72.6**	20.94
K_2O	14.2	15.4	1.3	0.8	0.3	0.4	0.5	1.3	14.5	0.5	1	0.9	1.4	15.4	0.6	16.5	0.6	0.8	**15.2**	0.90	**0.7**	0.41
CaO	3	3.6	0.6	0.6	3.6	0.7	0.8	1.8	2.2	1.1	0.8	0.4	0.7	3.2	0.6	3.6	1.5	0.9	**3.1**	0.58	**1.0**	0.87
Fe_2O_3	3.8	3.3	2.8	3.5	3.3	4.1	3.2	3.1	3.3	3.4	2.7	2.9	3.1	4.2	3.2	4.2	3.7	3.7	**3.8**	0.45	**3.1**	0.96
CoO	4.4	2.7	5.4	5.2	5.4	5.4	5.4	5.3	3.5	4.5	6.5	5.8	5.4	4.3	5.7	4	4.6	5.3	**3.8**	0.70	**5.0**	1.51
NiO	0.1	0.3	0.9	0.5	0.6	1	0.6	0.5	0.4	-	0.5	0.5	0.1	0.4	0.4	0.5	1	0.8	**0.3**	0.15	**0.5**	0.33
As_2O_3	6.3	5.2	8	9	7.2	5.6	6.6	8.2	5.5	9.2	8.3	8.5	8.5	5.9	8.3	7.1	8.2	8.8	**6.0**	0.74	**7.5**	2.35
Bi_2O_3	-	0.5	-	-	-	0.4	0.3	0.1	0.5	0.1	-	0.5	-	0.5	-	0.4	0.3	-	**0.4**	0.22	**0.1**	0.18
	100	100	100	100	100	100	100	100	100	100	100	100	100	100	100	100	100	100				

Table S4: Chemical composition of twenty-three smalt particles in sample SK-C-5_044. Values in yellow are deviating more than one standard deviation above the average and values in red are more than one standard deviation below the average. The well-preserved smalt particles are outlined with a black line.

SK-C-5_044	1	2	3	4	5	6	7	8	9	10	11	12	13	14	15	16	17	18	19	20	Average degraded	SD
Na_2O	0.4	0.2	0	0	0	0	0	0	0	0	0	0	0	0	0	0	0	0	0	0.1	**0.02**	0.05
MgO	0.1	0	0.6	0.2	0.5	0.2	0.2	0.4	0.5	0.4	0.2	0.4	0	0.2	0	0.3	0.3	0.3	0.1	0.1	**0.26**	0.18
Al_2O_3	1.4	2.2	2	3	1.7	1.5	1.8	2.5	2	2.2	2.4	1.4	2.3	2	2.3	2.1	2.2	1.9	1.6	2.1	**2.06**	0.38
SiO_2	75.4	77.3	84.6	76.9	80.7	80.2	83.9	79.6	78.8	78.7	77	86.5	77.8	80.4	77.6	80.3	84.7	78.8	77.6	78	**79.97**	2.91
K_2O	7.5	1.3	1.7	2.3	1.2	1.3	1.8	1.7	0.8	1.2	1.7	2.4	1.3	1.3	0.9	4.9	1.8	1.1	1.6	1.8	**1.69**	0.88
CaO	0.7	1.5	1.9	4.2	1.1	1.3	1.1	1.9	1.7	1.2	1.6	2.1	1.7	1.1	0.5	2.1	1.6	1.3	1.2	0.9	**1.58**	0.76
Fe_2O_3	3.2	2.8	2.5	3.6	2.9	2.5	3.1	3.5	2.9	3.3	3.3	2.7	3.1	2.6	2.9	3.6	2.7	3.1	3.9	2.9	**3.05**	0.40
CoO	4.1	4.9	2.3	3.4	3.3	4.7	1.9	3	4.9	4.4	4.5	2.6	4.4	4.4	4.8	3.1	1.7	5.2	4.5	5.2	**3.85**	1.14
NiO	0.4	0.1	1	0.6	1.1	1.3	0.8	0.9	0.5	0.1	0.5	0.4	0	0.5	0.4	0.8	0.6	0.4	0.3	0.2	**0.55**	0.36
As_2O_3	5.9	9.6	3.3	5.8	5.3	5.7	4.2	5.8	7.9	8.3	8.8	1.1	9.5	6.2	10.6	1.9	3.8	7.9	9.2	8.7	**6.51**	2.75
Bi_2O_3	0.9	0	0	0	2.3	1.3	1.1	0.8	0	0.1	0	0.6	0	1.1	0	0.8	0.8	0	0	0	**0.47**	0.65
	100	100	100	100	100	100	100	100	100	100	100	100	100	100	100	100	100	100	100	100		

Correlation cobalt and potassium

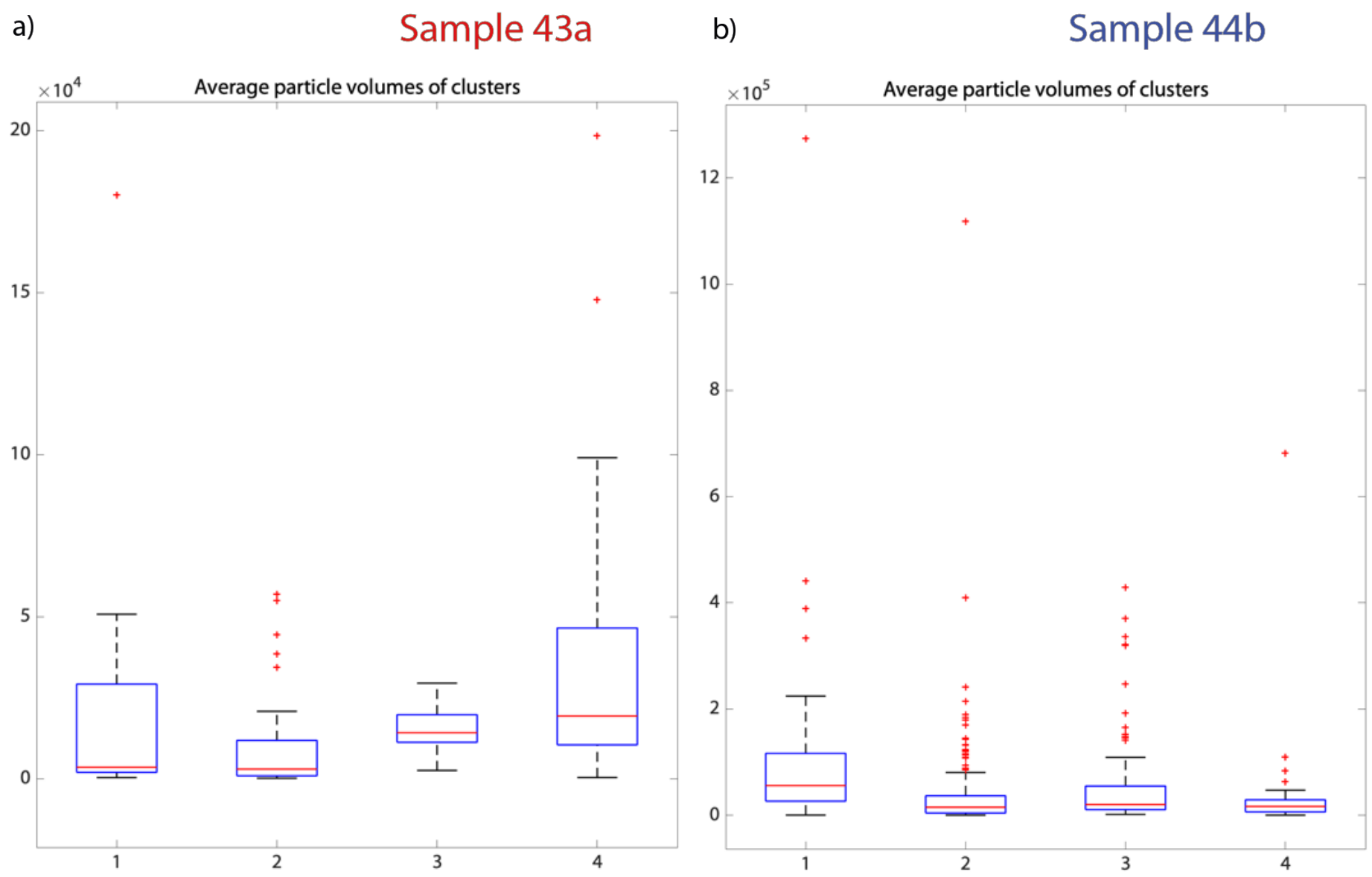

Figure S13: Average particle volume of the different clustering based on only the cobalt and potassium (multiplied by ten) intensities of samples (a) 43a and (b) 44b.

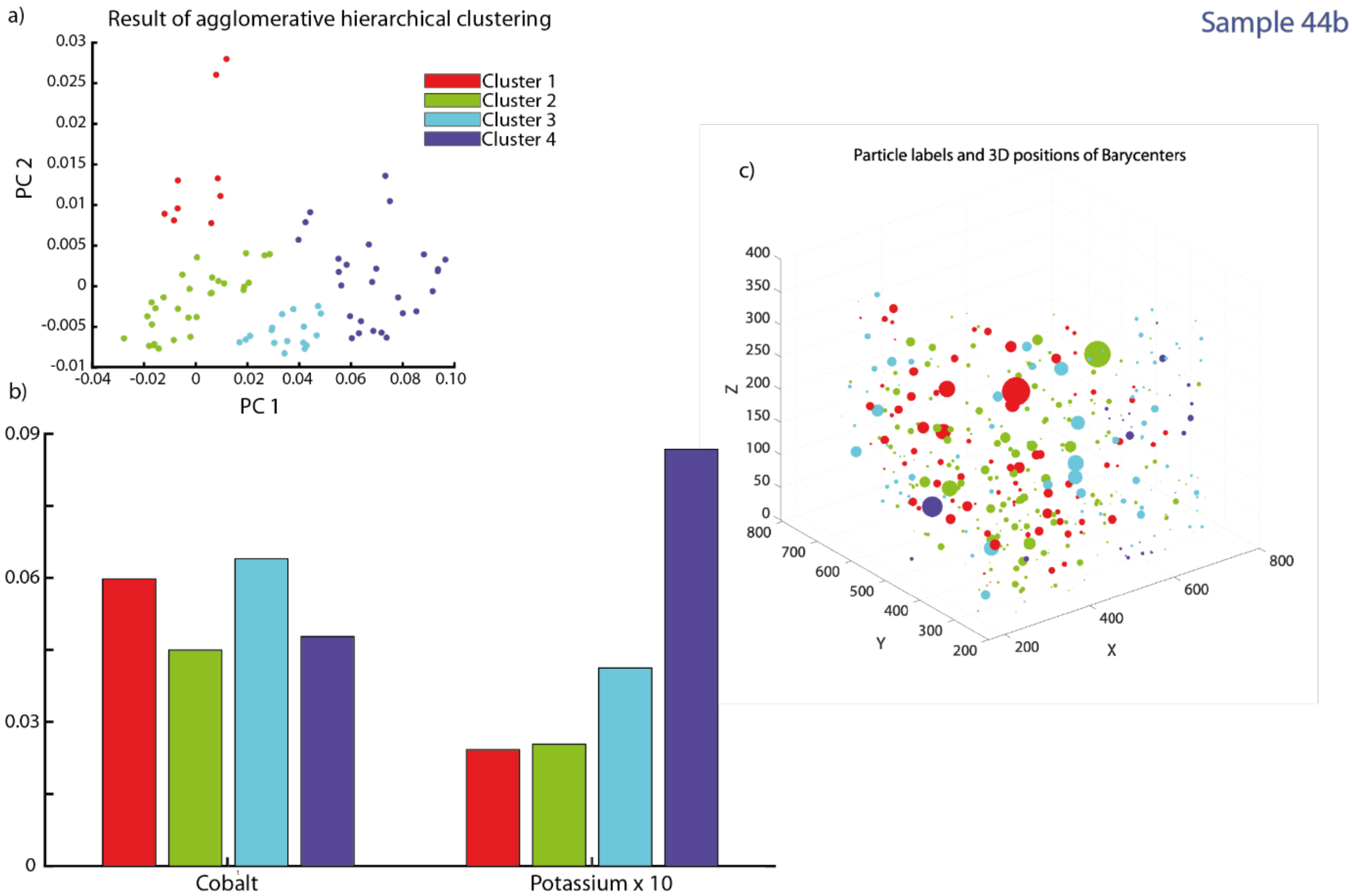

Figure S14: Results of the clustering of sample 44b based on only the cobalt and potassium (multiplied by ten) intensities. (a) Result of the agglomerative hierarchical clustering in PC1 and PC2 space. (b) average intensities of cobalt and potassium for the four clusters. (c) spatial distribution of the particles belonging to the four clusters. The size of the circles is proportionate to the volume of the particles they represent.

Additional figures

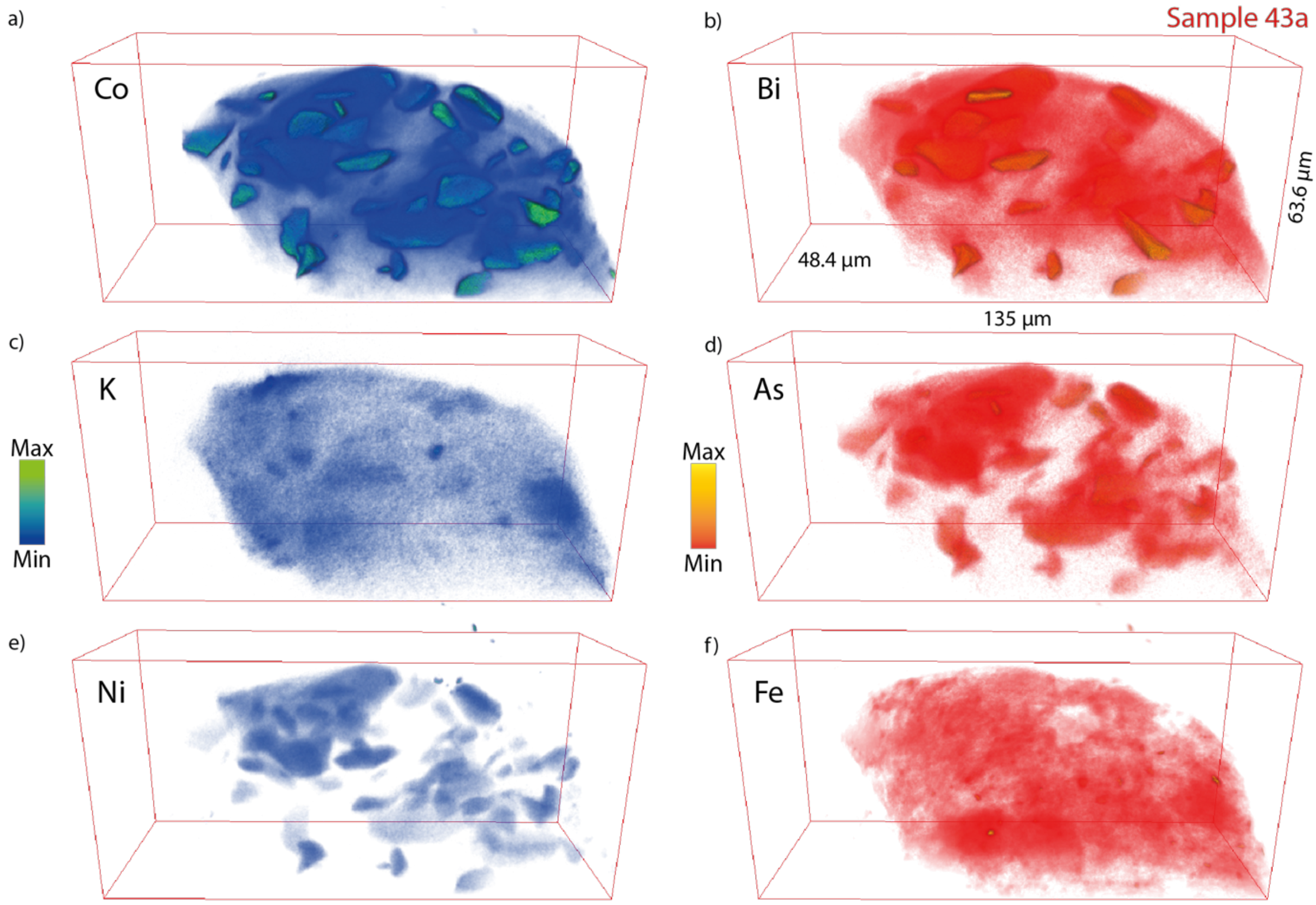

Figure S15. Volume rendering from the minimum to the maximum data of the elements related to smalt for sample 43a. (a) Cobalt (K), (b) Bismuth (L3), (c) Potassium (K), (d) Arsenic (Kb), (e) Nickel (Ni) (K) and (f) Iron (K).

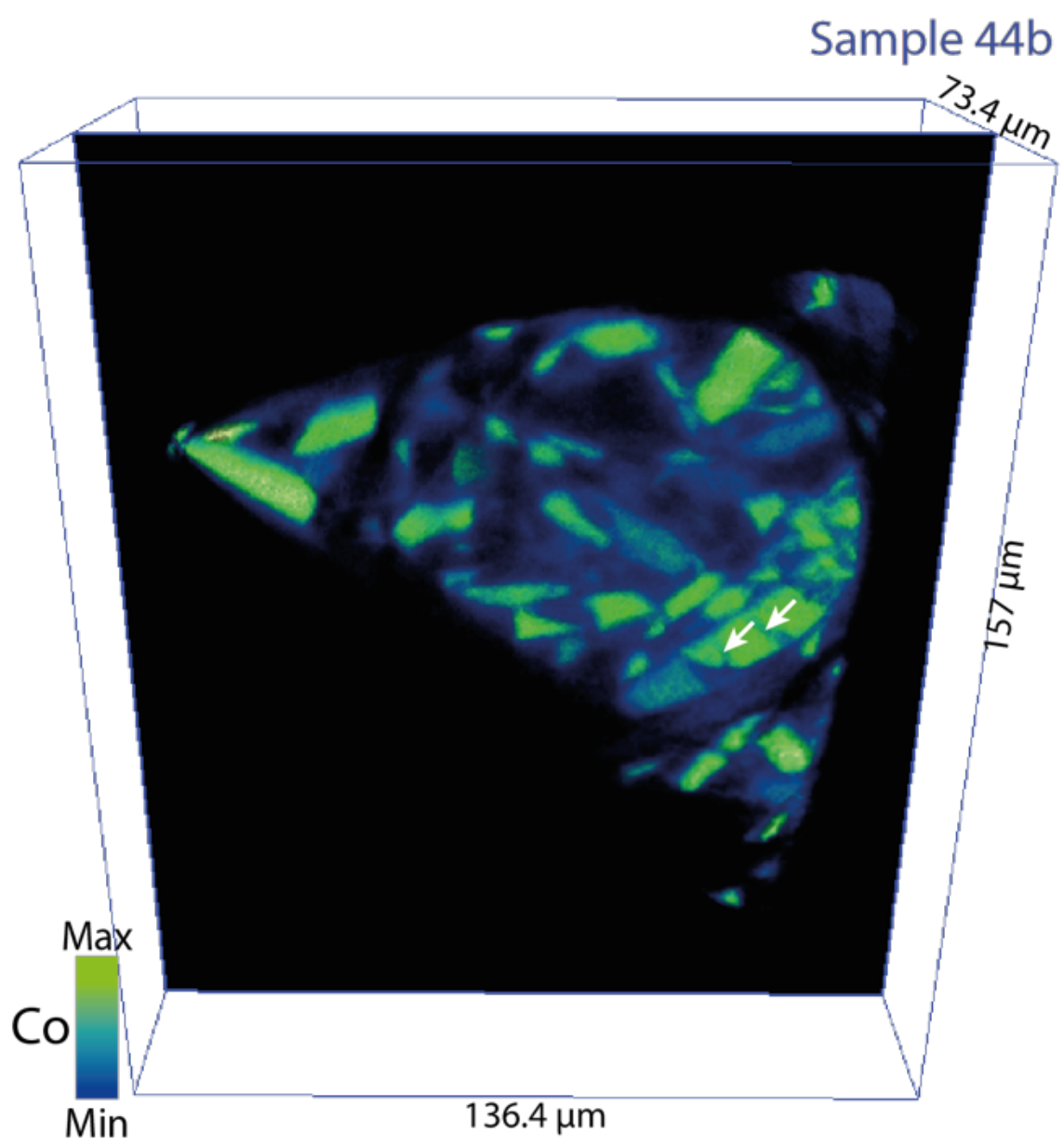

Figure S16. Microcracks (indicated by white arrows) in the middle of a smalt particle in sample 44b.

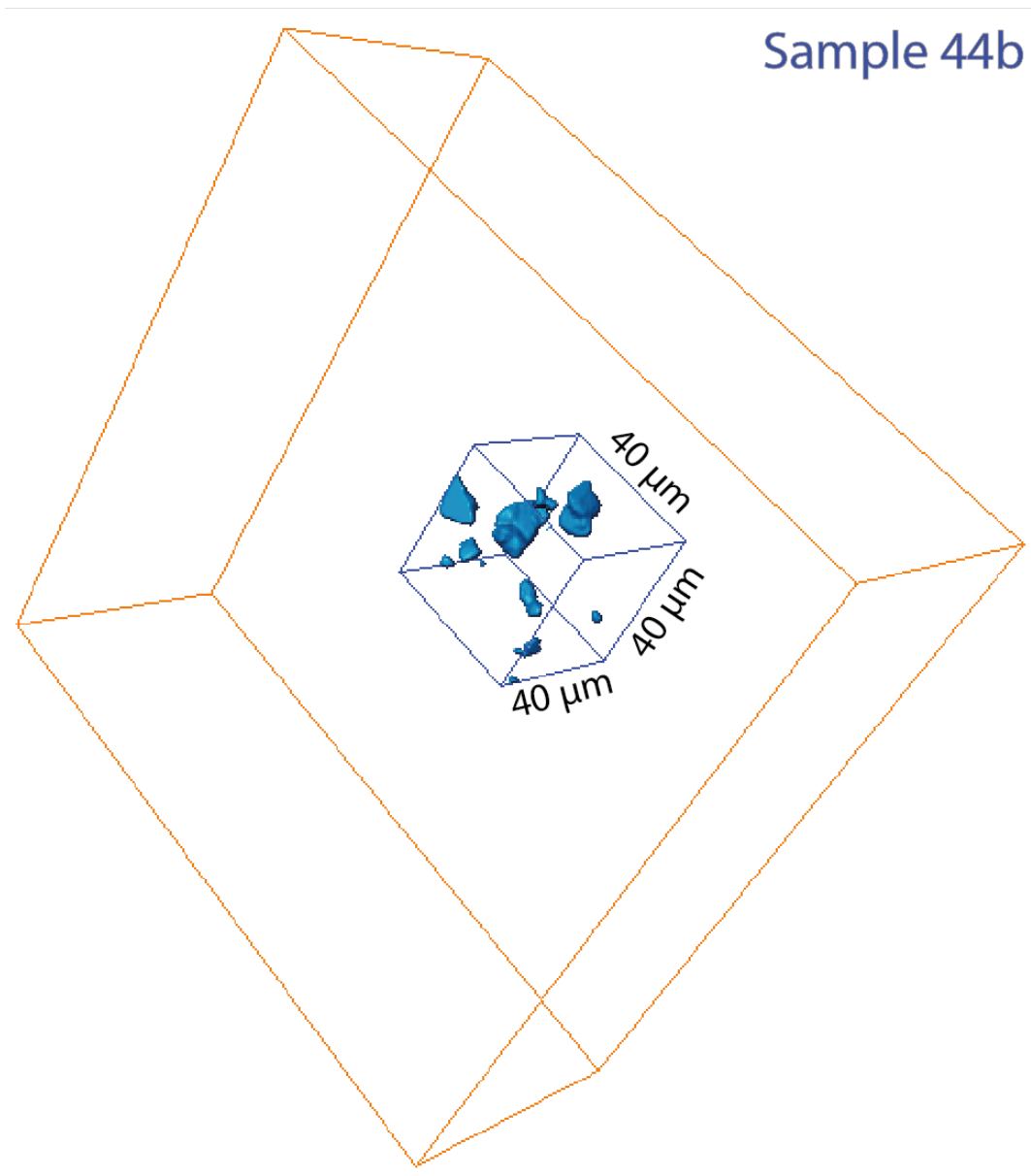

Figure S17. The sub volume (in blue) in sample 44 of 200 x 200 x 200 voxels that was used for the volume percentage calculations. The cyan particles visible here are the copper particles after thresholding.

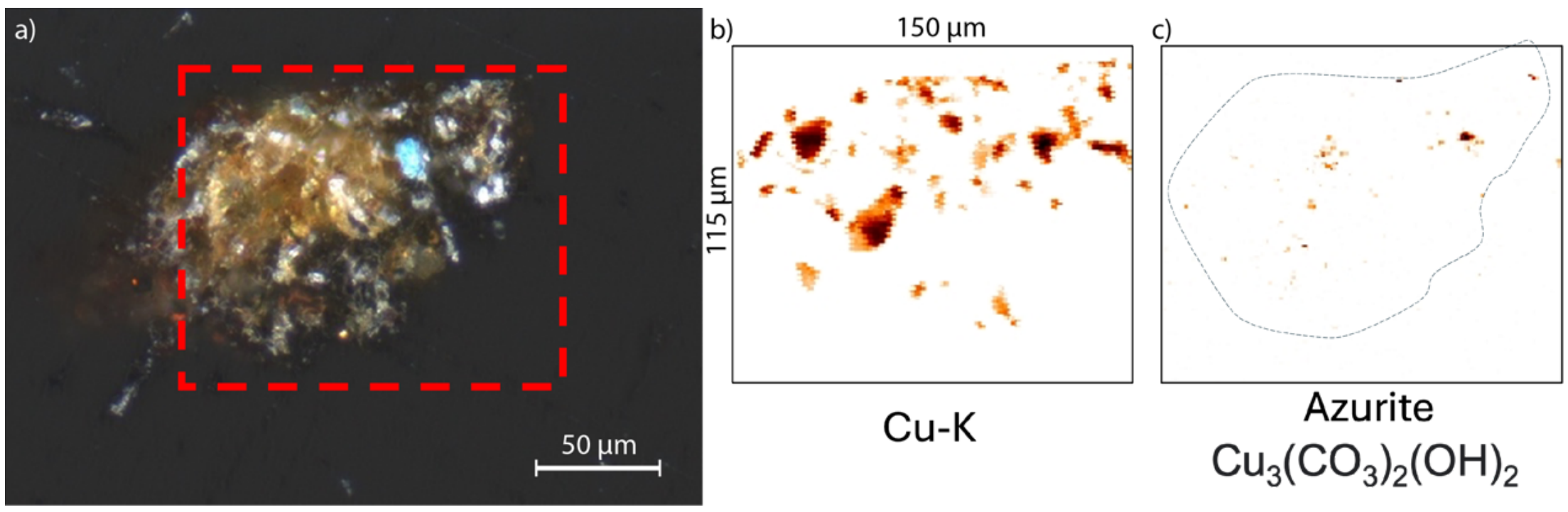

Figure S18. (a) Light microscopy image of sample 44b embedded (after tomography) red rectangle shows scanned area, (b) 2D µ-XRF copper distribution map and (c) µ-XRD distribution maps of azurite.

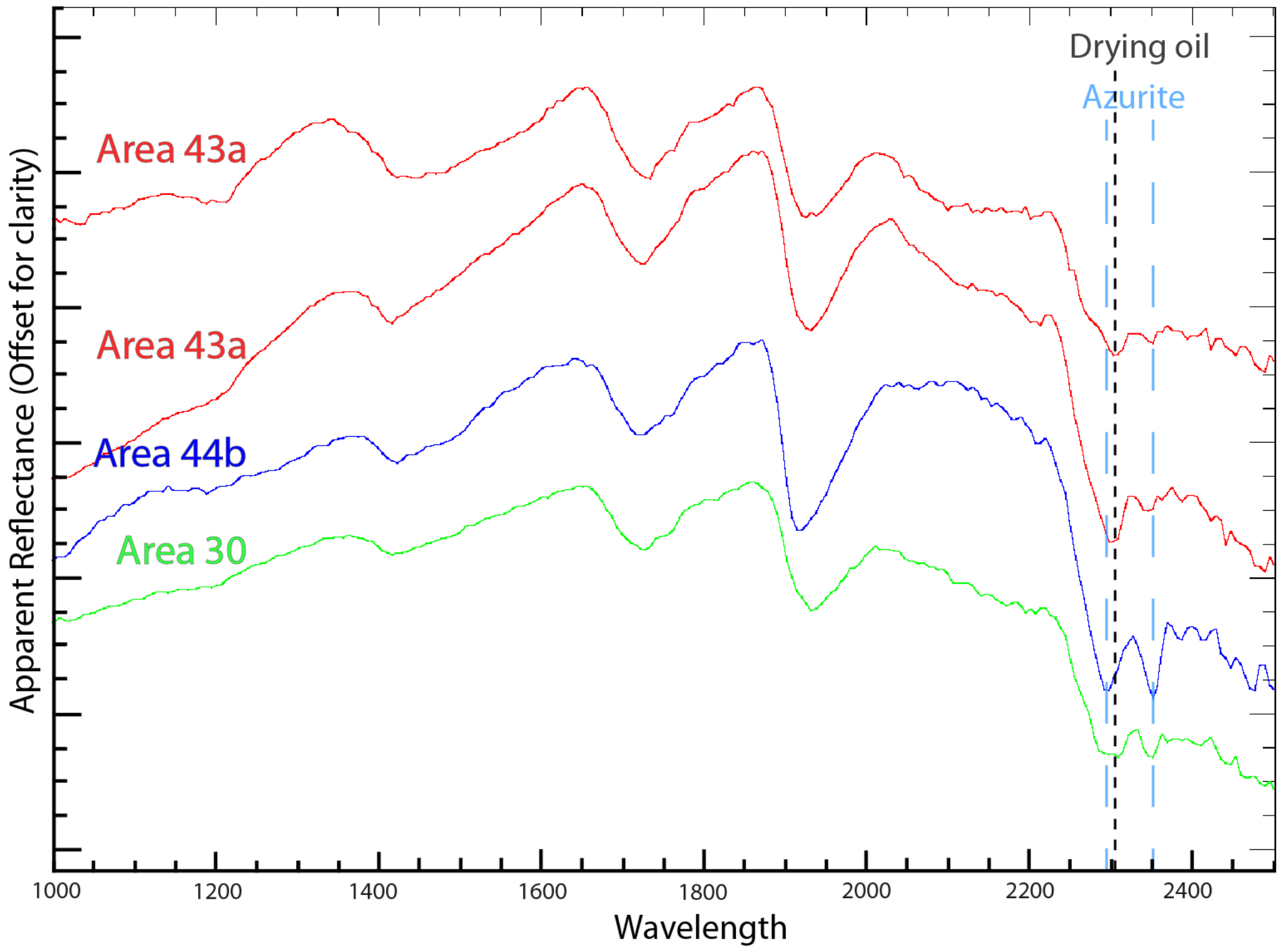

Figure S19. Reflectance spectra collected with short wave infrared reflectance imaging spectroscopy in the areas where samples 43a, 30 and 44b were taken. In the areas of 30 and 44b, the two strong, characteristic peaks of azurite are visible,[57] while these are missing in the two spectra collected in the area where sample 43a was taken. More information on the technique and instrument specifics can be found in the work by Gabrieli et al.[11]

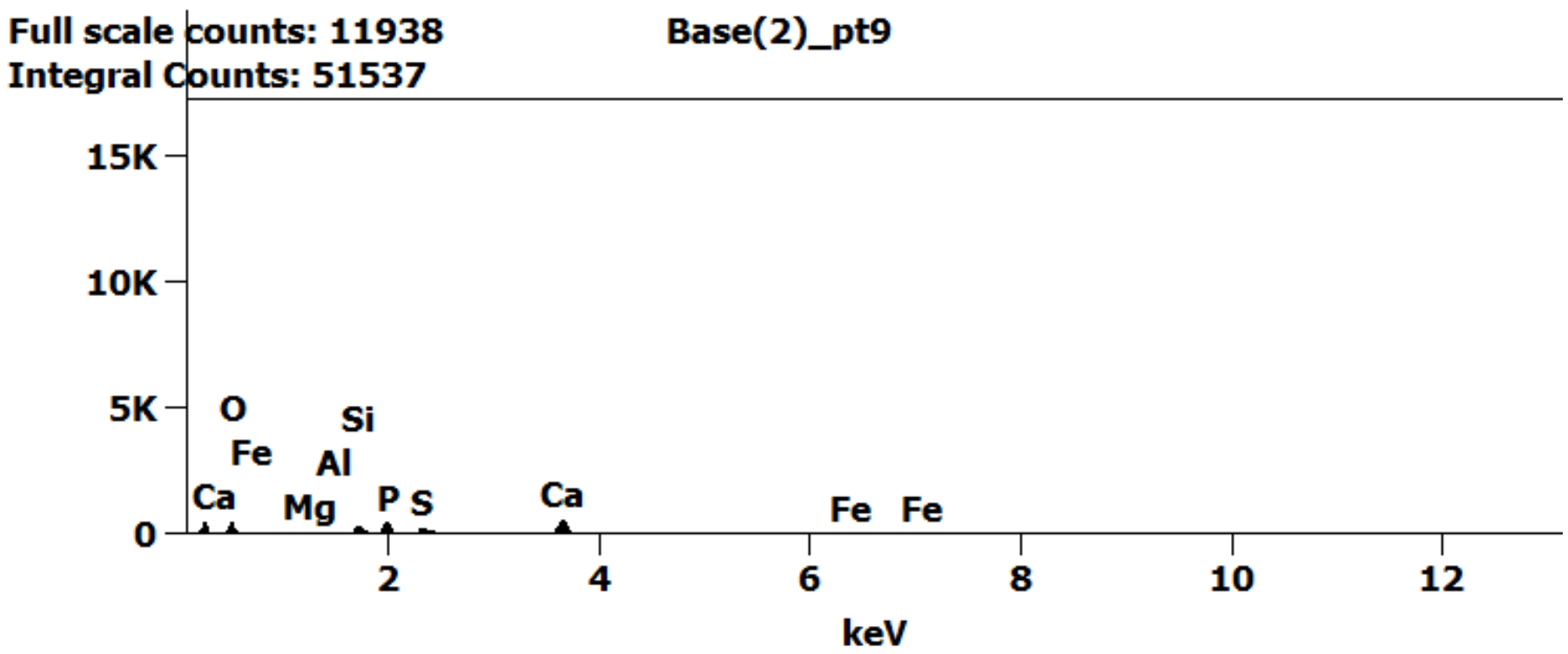

Figure S20. SEM-EDX spectrum collected in a bone black ($Ca_5(OH)(PO_4)_3$ and carbon) pigment particle in sample 43a, showing the presence of both Ca and P.

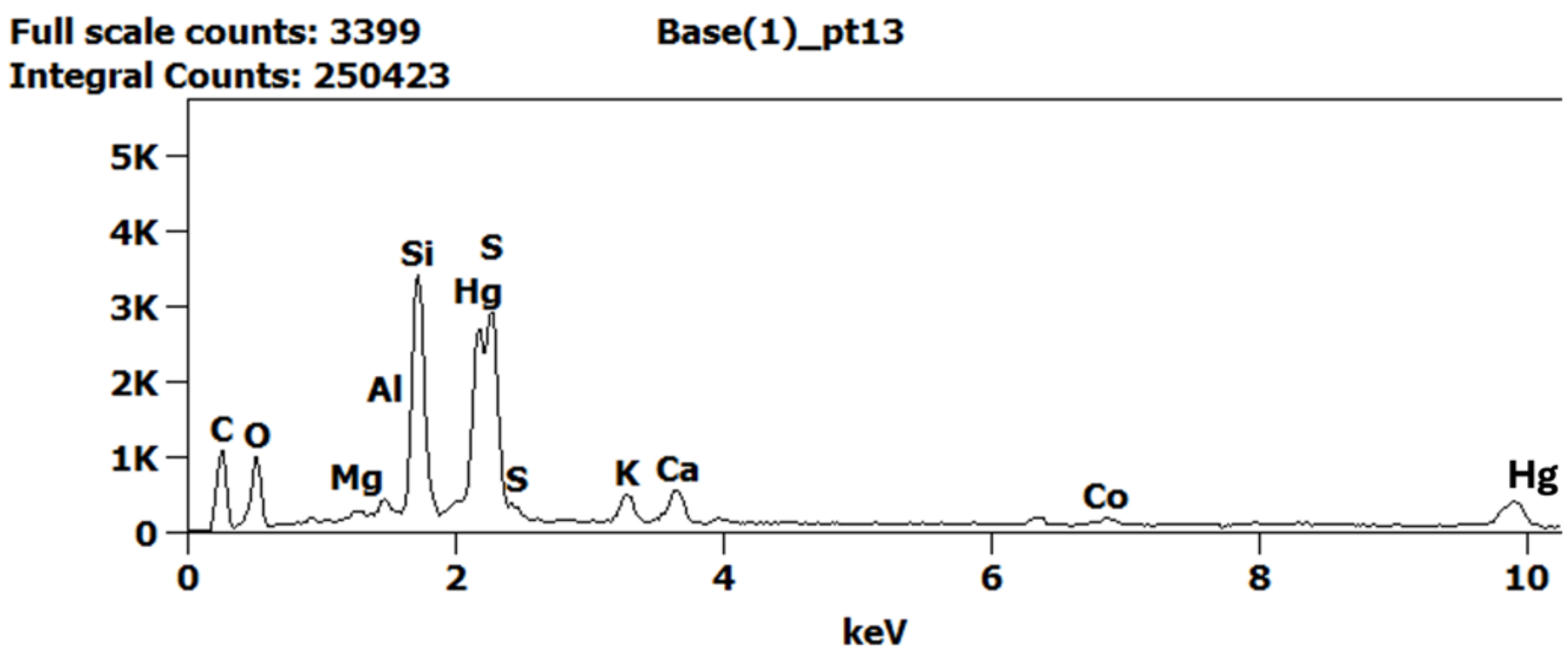

Figure S21. SEM-EDX spectrum collected in mercury (HgS) pigment particle in sample 44b, showing the presence of Hg and S.

Estimation of spatial resolution of the 3D reconstructions

The achieved spatial resolution in 3D can be estimated by fitting a Gaussian profile over the change in intensity of the grey values along the line profiles of a sharp edges in one of the ortho slices. This was done by using an in-house developed MATLAB script.[43,58]

Figure S4.15 shows the spatial resolution estimation of the XRF results, calculated on sharp edges within ortho slices of the cobalt and copper (Figure S4.14). The resolution of samples 43a and 44b are both around 670 nm, and the resolution of sample 30 is 916 nm. This lower resolution of sample 30 is probably due to the fact this sample was embedded when measured. Additionally, some artifacts were observed in the projections, probably due to motor movements during the scans.

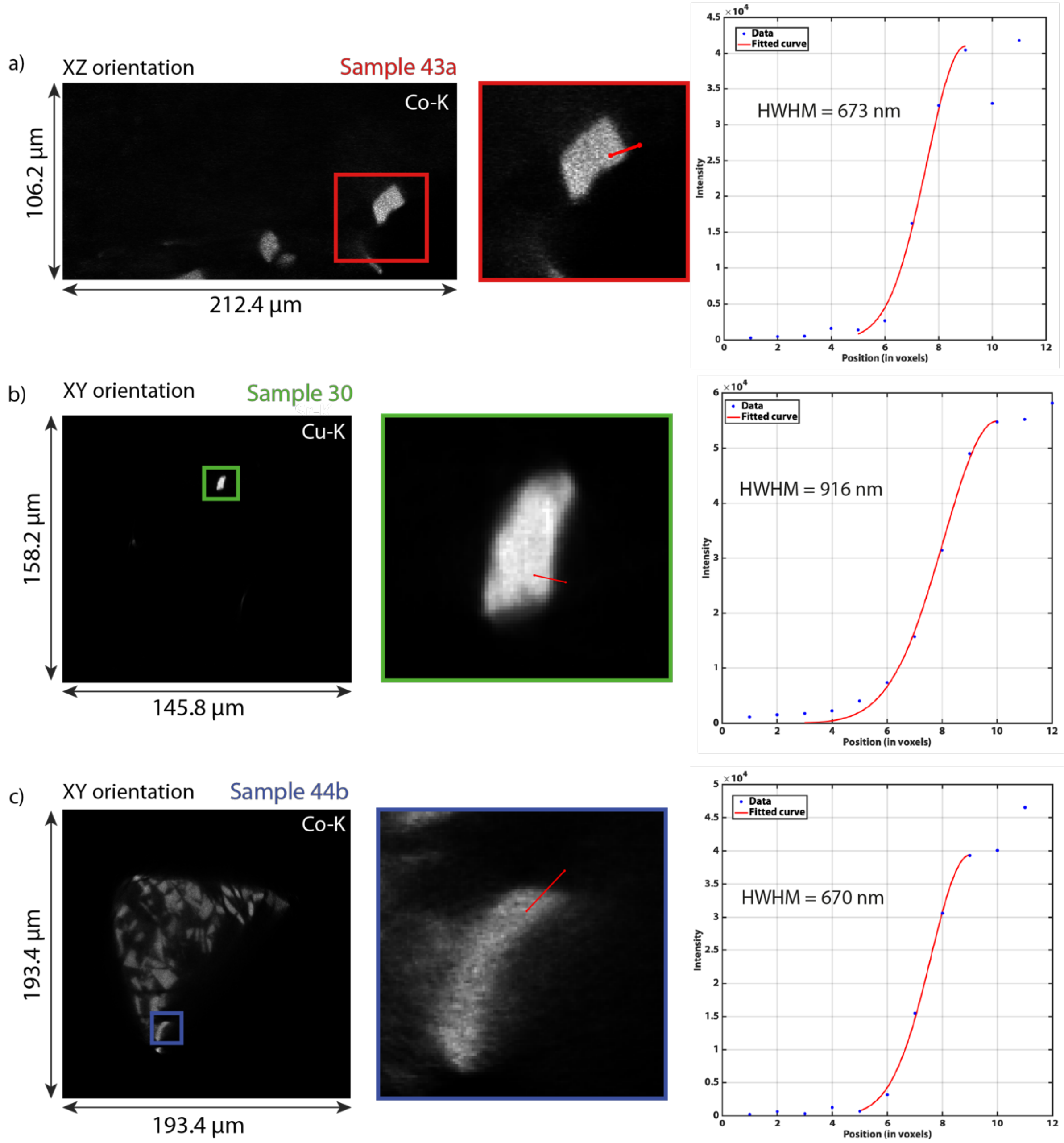

Figure S22. Reconstructed orthoslices of the data sets (voxel size 200 nm). The boxes show a zoom-in of the orthoslice where a line profile was measured (red line). On the right the data points are shown with the fitted Gaussian to these points. The half width half maximum (HWHM) is the measure for the estimation of the achieved resolution of the 3D dataset. (a) sample 43a using the of the Co-K distribution resulting in an estimated resolution of 673 nm, (b) sample 30 using the Cu-K distribution resulting in an estimated

resolution of 916 nm and (c) sample 44b using the Co-K distribution resulting in an estimated resolution of 670 nm.
